# Reduction of false alarms in the intensive care unit using an optimized machine learning based approach

**DOI:** 10.1038/s41746-019-0160-7

**Published:** 2019-09-05

**Authors:** Wan-Tai M. Au-Yeung, Ashish K. Sahani, Eric M. Isselbacher, Antonis A. Armoundas

**Affiliations:** 1 Cardiovascular Research Center, Massachusetts General Hospital, Boston, MA 02114 USA; 2 Healthcare Transformation Lab, Massachusetts General Hospital, Boston, MA 02114 USA; 3 Institute for Medical Engineering and Science, Massachusetts Institute of Technology, Cambridge, MA 02139 USA

**Keywords:** Health care, Biotechnology, Biomedical engineering

## Abstract

This work attempts to reduce the number of false alarms generated by bedside monitors in the intensive care unit (ICU), as a majority of current alarms are false. In this study, we applied methods that can be categorized into three stages: signal processing, feature extraction, and optimized machine learning. At the stage of signal processing, we ensured that the heartbeats were properly annotated. During feature extraction, besides extracting features that are relevant to the arrhythmic alarms, we also extracted a set of signal quality indices (SQIs), which we used to distinguish noise/artifact from normal physiological signals. When applying a machine learning algorithm (Random Forest), we performed feature selection in order to reduce the complexity of the models and improve the efficiency of the algorithm. The dataset used is from Reducing False Arrhythmia Alarms in the ICU: the PhysioNet/Computing in Cardiology Challenge 2015. Using the performance metric “score” from the Challenge, we achieved a score of 83.08 in the real-time category on the hidden test set, which is the highest in all published work.

## Introduction

In the intensive care unit (ICU), bedside monitors are used to alert healthcare providers when a patient’s physiological signals are out of normal range so that an appropriate response can be provided. In a prior study, it was discovered that 88.8% of annotated arrhythmia alarms were false positives.^1^ Therefore, the majority of alarms do not require clinical intervention and, consequently, become a burden.^1,2^ Excessive numbers of false alarms cause noise disturbance (the volume is often over 80 dB in an ICU^3,4^), desensitization, and decreased quality of care, such that false alarms have often been listed as one of the top technology hazards.^5,6^

Common sources of false alarms in the ICU are noisy physiologic signals that go out of range. Many attempts have been made to alleviate the problem of false alarms, including sensor fusion methods using multiple physiological signals^7^, signal processing methods (such as median filters) to improve the signal quality^8^, and artificial intelligence methods (such as rule-based expert systems).^9^ For example, an algorithm to suppress false critical electrocardiographic (ECG) arrhythmia alarms using morphological and timing information using the arterial blood pressure (BP) signal was proposed in a study by Aboukhalil et al.^10^ That algorithm was able to suppress 59.7% of the false alarms while the true alarm reduction rates were all 0%, except for ventricular tachycardia alarms at 9.4%. In another study, Li et al.^11^ presented a framework for false alarm reduction using a machine learning approach that combined up to 114 signal quality and physiological features extracted from the ECG, photoplethysmograph (PPG), and, optionally, the BP waveform. In that study, false alarm suppression rates were 86.4% for asystole, 100% for extreme bradycardia, 27.8% for extreme tachycardia, and 19.7% for ventricular tachycardia, with 0% true alarm suppression. Although these methods are promising, much improvement is still needed.

In this study, we aimed to achieve a high false alarm suppression rate with a low true alarm suppression rate by utilizing features that characterize the arrhythmias and quantify the signal quality, and an optimized machine learning based approach. The features include a set of signal quality indices (SQIs) that can distinguish noise/artifact from normal physiological signals. The introduction of the SQIs is inspired by the fact that the source of many false alarms is noise/artifact in the physiological signals.^9^ If noise/artifact can be distinguished from normal physiological signals reliably using these SQIs, the number of false alarms could be greatly reduced. Also, we utilized a machine learning-based method as it is capable of finding an underlying structure in a complex dataset.^12^ Since, in the ICU, decisions about whether or not to sound an alarm need to be made in real time, reducing the complexity of the models and increasing their efficiency by selecting an optimal subset of features makes the use of machine learning algorithms an appealing approach. We validate these algorithms in a dataset from the PhysioNet 2015 Challenge (physionet.org) that offers 1250 true or false alarms separated into a training set of 750 alarms and a test set of 500 alarms. For each alarm, the signals provided are ECGs, and/or PPG, and/or BP. The alarms were annotated as true or false by a team of expert annotators.

## Results

### Determining the cost of false negatives

Supplementary Figs. 1–5 show that the classification performance vs. the cost of false negatives (FN) for each type of arrhythmia, while the cost of false positives (FP) is fixed at 1. It can be observed that as the cost of FN increases, the true positive (TP) rate mostly increases while the true negative (TN) rate mostly decreases for all types of arrhythmia. This is because the machine learning algorithm is trying to minimize the total cost of errors. As the cost of FN increases, the machine learning algorithm tries to classify more positive records correctly while sacrificing the accuracy of negative record classification. Supplementary Table 1 shows the cost of FN chosen for each type of arrhythmia, which maximize the overall score. They are all in the range of 1–1.4.

### Feature selection for each arrhythmia

Supplementary Figs. 6–10 show the plot of importance of features for each type of arrhythmia.

Asystole–for asystole, the most important feature is ECG 1 maximum RR interval between consecutive R-waves. Besides maximum RR interval between R-waves, one can see that different measures of swing play important roles in the classification performance as well.

Bradycardia–for bradycardia, the three most important feature is the minimum heart rate measured from the ECG signals and the PPG.

Tachycardia–for tachycardia, the two most important feature is the maximum heart rate measured from the ECG signals, followed by the SQIs for ECGs which include correlation measure, peak height stability measure and sharpness measure.

Ventricular fibrillation–for ventricular fibrillation (VF), frequency domain features of the ECG signals, are the most important ones: the mean frequency, the median frequency, and the maximum power to the total power ratio. This is because during VF the ECG resembles a sinusoidal signal. Frequency domain analysis presents a simple yet effective way to separate true VF from false VF.

Ventricular tachycardia–for ventricular tachycardia (VT), the most important feature is the ECG correlation measure, which makes sense as consecutive beats have a uniform and stable QRS morphology during monomorphic VT. Two other SQIs—peak height stability measure and periodicity measure—are the next most important features.

### Random forest and feature selection

Supplementary Figs. 11–15 show the median and mean score curves vs. the number of features selected for each type of arrhythmia. The most important feature was used, as the predictor, first, and then the next most important feature was added as a predictor, one by one. For each type of arrhythmia, it can be observed that the mean and median scores that measure the performance of the classification began to plateau before all the features were used as predictors. Therefore, we can reduce the number of features used for building the classifiers but still retain the same level of performance. Table 1 shows the total number of features considered, in the initial selection and in the final selection for each type of arrhythmia. Supplementary Tables 2–6 list the features selected in the final selection with their importance, in descending order. Supplementary Figs. 16–20 show the scatter plot of true and false alarms with the two most important features for each type of arrhythmia.

**Table 1:** Number of features considered, in the initial selection and in the final selection for each type of arrhythmia

Arrhythmia	Total number of features considered	Number of features in the initial selection	Number of features in the final selection
Asystole	35	20	25
Bradycardia	22	14	18
Tachycardia	22	12	15
Ventricular fibrillation	32	23	29
Ventricular tachycardia	28	23	28

### Algorithm comparison with the state of the art

As shown in Table 2, the results on the hidden dataset are good for all types of arrhythmias, except bradycardia, compared with published results^13–16^. Notably, we achieved the highest scores in all published studies in tachycardia, VF, and VT. After obtaining the test result presented in Table 2, we attempted to improve our algorithm's performance to classify bradycardia alarms.

**Table 2:** Result with the hidden test set

	TPR (%)	TNR (%)	Score
Asystole	94	93	91.19
Bradycardia	74	74	52.55
Tachycardia	100	100	100
Ventricular fibrillation	100	92	93.10
Ventricular tachycardia	88	86	78.67
Real-time	92	87	79.89
Retrospective	93	89	82.12

By visualizing the vital-sign signals of the bradycardia alarms, we have observed that there is at least one reliable signal in each of these alarms. For example, as seen in Supplementary Fig. 21, the reliable signal is the ECG signal, while the PPG signal looks very noisy. As a result, for the final implementation, the most reliable signal was chosen by selecting the signal with the highest correlation measure for bradycardia. Then, its slowest rate for 4 consecutive beats was calculated: If the rate was slower than 46 bpm, then the alarm was classified as true; otherwise, the alarm was classified as false. After changing the method of classification of bradycardia alarms, the classification performance of bradycardia alarms improved (Table 3), and resulted to the highest overall score.

**Table 3:** Result with the hidden test set after changing the method for classification of bradycardia alarms

	TPR (%)	TNR (%)	Score
Asystole	94	93	91.19
Bradycardia	97	62	73.27
Tachycardia	100	100	100.00
Ventricular fibrillation	100	92	93.10
Ventricular tachycardia	88	86	78.67
Real-time	95	85	83.08
Retrospective	98	87	87.60

## Discussion

A high volume of false alarms in the ICU creates a noisy environment and causes alarm fatigue among caregivers. Many efforts have been made to reduce the number of false alarms, but clearly better solutions are still needed. In this report, we present a method that aims to reduce false alarms in the ICU, that is based on signal processing, feature extraction, and machine learning tools. Moreover, SQIs, such as correlation measure, are introduced. Several conclusions can be drawn from this study: first, domain knowledge is important in feature design as the features introduced in this paper, especially the SQIs, are based on an understanding of physiological signals, and they play an important role in the classification performance; second, adjusting the ratio of misclassification cost of FNs and FPs helps optimize the performance of the machine learning algorithms; third, redundant features may be eliminated by forward feature selection, which would lead to simpler, more efficient, and equally accurate (if not more accurate) machine learning models; and, fourth, a combination of features with good discriminating power and modern machine learning algorithm can help reduce the number of false alarms.

The proposed method achieved a higher score than any of the previously published methods that have used the PhysioNet 2015 challenge hidden test set. Machine learning algorithms such as Support Vector Machine and Random Forest have been employed by some of the competitors in the PhysioNet/Computing in Cardiology 2015 Challenge, but they did not achieve the same level of performance.^14,15,17^ This may be due to the quality and the quantity of features we used. Domain knowledge is important in feature selection and design. In this work, we devised the features based on understanding of cardiovascular signals and human physiology. Different features that characterize the arrhythmias, such as blank area swing used for asystole alarms or maximum power to total power ratio used for VF alarms, have been pivotal in the performance of the classification. Also, although SQIs are not directly related to the characteristics of the different arrhythmias, they have played an important role in the classification of the alarms and that was shown by their importance calculated with the Random Forest (RF) algorithms.

In the proposed algorithm, all features including the SQIs and the arrhythmia-specific features were fed into the RF classifiers at the same stage. Given the good classification performance, it can be concluded that the RF classifiers are able to separate true alarms and false alarms using the SQIs and arrhythmia-specific features, without the need of further post-processing of these features before feeding them into the RF classifiers. It should be noted that a previous study also used RF as its machine learning algorithm.^14^ However, one major difference between our approach and that study is that we input all the arrhythmia-specific features and SQIs from all available signals into the RF classifier, while the previous study performed signal selection by their purity first, and then input features from these selected signals into the RF classifiers.

Another reason for our method’s good performance is that, in order to maximize the score, we tuned the ratio of the cost of the FN to FP for each type of arrhythmia. One can almost always improve the overall performance by tuning the hyper-parameters of the machine learning algorithms. Very lengthy analysis is often required to determine the true ratio of the cost of FP to FN, as there are many different factors to be considered, including the risks patients encounter as a consequence to false negative alarms and the desensitization the caregivers experience due to false positive alarms. Although we did not perform such an analysis in the current report, we set the ratio to give the maximum score in this specific dataset.

The fact that the algorithm performed less well than expected in classifying bradycardia alarms in the hidden test set may be explained by the fact that the training and hidden test sets differ significantly in terms of bradycardia events. We managed to improve the score for bradycardia by choosing the most reliable signals and comparing their correlation measures. Similar methods estimating the heart rate using multiple signals have been developed before.^10,18^

Because our ultimate goal is to implement such algorithms in real time, we performed feature selection. As the algorithms are more efficient when fewer features are calculated, we managed to reduce the number of features used in the final models for asystole, bradycardia, tachycardia and ventricular fibrillation, while not compromising the performance of the RF classifiers, as shown in Supplementary Figs. 11–15.

The dataset used in this study is relatively small, especially for VF, for which there are only 6 true alarms. In the future, our algorithms should be trained, validated, and tested using larger datasets to examine if they would perform at a similarly high level. In addition, challenges remain before our algorithms can be implemented in clinically active bedside monitors. For example, the TP rates are still below 100% for some types of arrhythmias, which means that some of the true alarms will be missed. Missed alarms can have significant consequences, including even patient death. Although investigators have made great efforts to reduce the rate of false alarms in the ICU through improvement in the arrhythmia detection algorithms, perfect results (i.e., TP rate = 100% and TN rate = 100%) have never been achieved on any datasets, confirming how challenging this problem is. In the future, rather than solving this problem by improving the arrhythmia detection algorithms alone, supplemental approaches could also be introduced to manage ICU alarms more effectively, such as alarm training and prioritizing actionable alarms.^19^

In this work we have used SQIs, arrhythmia-specific features and and an optimized machine learning approach to classify ICU arrhythmia alarms and, in doing so, we have achieved the highest score among all published works in the hidden test set from the PhysioNet Challenge 2015. This demonstrates that excellent classification results can be achieved with good feature engineering and the use of an advanced machine learning algorithm. Such an approach therefore has the promise to improve the ICU environment for patients and healthcare providers alike.

## Methods

### Dataset

The dataset we used is from the Reducing False Arrhythmia Alarms in the ICU: the PhysioNet/Computing in Cardiology Challenge 2015.^13^ This challenge used bedside monitor data with a total of 1250 life-threatening arrhythmia alarms recorded from three of the largest intensive care monitor manufacturers’ bedside units. These alarms occurred because the monitors detected the occurrence of either asystole, extreme bradycardia, extreme tachycardia, ventricular tachycardia or ventricular flutter/fibrillation. The alarms were annotated as true or false by a team of expert annotators according to the definitions listed in Table 4. These alarms were divided into a training set and a test set. These training and test sets consist of two subsets of mutually exclusive patient populations. The training set has 750 recordings and it is publicly available while the test set has 500 recordings and it is hidden from the public. All the alarms occurred at the 300th second of the records. Due to the retrospective nature of this study using only publically available data, ethics approval for the study was not required.

**Table 4:** Definition of the five types of arrhythmia

Arrhythmia	Definition
Asystole	No QRS for at least 4 s
Extreme bradycardia	Heart rate lower than 40 bpm for 5 consecutive beats
Extreme tachycardia	Heart rate higher than 140 bpm for 17 consecutive beats
Ventricular tachycardia	5 or more ventricular beats with heart rate higher than 100 bpm
Ventricular flutter/fibrillation	Fibrillatory, flutter, or oscillatory waveform for at least 4 s

### Performance metrics

The four possible outcomes of the classification algorithm are TP, TN, FP, and FN as illustrated in the confusion matrix in Supplementary Table 7. The performance of all classification algorithms is further quantified by the TP rate, TN rate and score set by Computing in Cardiology. These three metrics are described by Eqs. (1–3) respectively:

1 $${\mathrm{TP}}\,{\mathrm{rate}} = \frac{{{\mathrm{TP}}}}{{{\mathrm{TP}} + {\mathrm{FN}}}}$$

2 $${\mathrm{TN}}\,{\mathrm{rate}} = \frac{{{\mathrm{TN}}}}{{{\mathrm{TN}} + {\mathrm{FP}}}}$$

3 $${\mathrm{Score}} = \frac{{{\mathrm{TP}} + {\mathrm{TN}}}}{{{\mathrm{TP}} + {\mathrm{TN}} + {\mathrm{FP}} + 5 \times {\mathrm{FN}}}} \times 100$$

Note that in the denominator of the Score, FN is multiplied by 5 compared to FP. This makes clinical sense as a missed alarm (FN) is of greater consequence than a FP.

### Signal processing

#### Modified Zong’s method for identification of valleys of BP and PPG

Zong et al. had reported an open source algorithm for the identification of the onset of BP pulses.^20^ Building upon Zong’s method, we calculated the slope sum function (SSF) twice on the signal and skipped the low-pass filtering step. This method was applied to both BP and PPG signals; S1 = SSF(BP or PPG), S2 = SSF(S1).

S2 has very sharp peaks that are similar to the QRS complex on the ECG. The peaks of S2, which indicate the onset of the waveform in the original signal, are then detected by using Martínez’s method for QRS detection.^21^ The advantage of this method is that it works well for both BP and PPG and it is insensitive to baseline wander, which can be very close to the heart rhythm. Plots of BP and its double SSF were shown in Supplementary Fig. 22.

#### Signal abnormality of the BP waveform

First, we identified good quality portions of the BP signal by using all criteria proposed by Sun et al.,^22^ except the heart rate and the change of the duration of successive beats. Then, we extracted features from the good-quality BP signals for the purpose of classification of the alarms as true or false.

#### Flat line detection

No processing is done if there is a flat line within a window. A signal window is said to contain a flat line if it contains a constant value for at least 2 seconds. This indicates probe disconnection.

#### Baseline wandering removal for ECG

As a preprocessing step for analyzing the ECG signals, we remove the baseline wandering from the signals. This is done with modeling the ECG segments within the windows with a 5th order polynomial. This is effective for removing most of the baseline wandering.

#### R-wave peak detection of the ECG signals

After baseline wandering is removed, we use a state-of-the-art ECG delineation algorithm designed by Martínez et al. based on the wavelet transform (WT).^21^ The method has been reported to yield over 99.5% sensitivity and positive predictive value in identifying the QRS complex in standard ECG databases. Supplementary Fig. 23 shows a plot of ECG and the R-wave peak detections.

#### Amplitude envelope estimation of the ECG signals

The ECG delineation algorithm can result in false R-wave peak detection due to artifact. We adopted the method of amplitude envelope estimation proposed by Plesinger et al. to mitigate this problem.^16^ This method can be used to detect false R-wave peaks due to high-frequency pacing spikes and T-wave over-sensing. In addition, it can be used to identify ventricular tachycardia (VT) beats. Supplementary Figs. 24–26 show representative examples.

#### Feature extraction

Feature extraction was performed on ECG, BP, and PPG signals. Classifiers were built using these features and the expert annotations as inputs. We extracted a set of signal quality indexes (SQIs) on all records and relevant features based on the definition of the arrhythmias. Supplementary Table 8 shows the number of seconds each record was analyzed for each type of arrhythmia. Feature extraction is performed only once for each record, and each record is represented by one vector of features except for true alarms in VF. We performed feature extraction four times for each true VF alarm starting at the end of 293rd second, 294th second, 295th second, and 296th second. The onset of VF must be within 10 s of the alarm in order to meet the American National Standards Institute/Association for the Advancement of Medical Instrumentation (ANSI/AAMI) EC13 Cardiac Monitor Standards, which means that the four seconds of signals that trigger the VF alarms may not be starting at the end of the 296th second. The descriptions of the features can be found in the Supplementary Methods. Illustrations of a subset of features can be found in Supplementary Figs. 27–35.

#### Machine learning–random forest algorithm

The machine learning algorithm used, was Random Forest (RF),^23,24^ in MATLAB 2016B using the function TreeBagger. RF is an ensemble learning method that can be used for classification. It grows a multitude of decision trees during training time, and each decision tree is trained with a bootstrap sample.^25^ At each split of the decision tree, a number of features, which is set to be equal to the square root of the total number of features, are randomly selected. From these randomly selected features, those that, based upon the Gini’s diversity index^26^, best separate the true from false alarms, are used to create the split. For classification, each decision tree makes a vote and the final result of classification is the mode of all the votes. We set the number of trees to be equal to 301. At first, the default settings in the MATLAB 2016B TreeBagger function were used to create the RF. However, while attempting to optimize our algorithm, we realized that by changing the settings—such as the misclassification costs of true and false alarms—could improve the final performance on the hidden test set. A more detailed description of how we obtained the optimal misclassification costs is provided below, in the cost-sensitive learning section.

RF was selected because it gave the best result compared to support vector machines and shallow neural networks when these three algorithms were evaluated on the training set. More information about the performance of these machine learning algorithms can be found in the Supplementary Table 9.

#### Cross-validation on the training set

As our goal was to achieve good classification in the Physionet hidden data set, we performed cross-validation on the training set to estimate the performance of our algorithm on unseen data. Due to the small sample size, the leave-one-out cross validation was performed.^27^

#### Feature ranking and selection

We measured and ranked the importance of features by examining the increase of the prediction error if the values of that variable are permuted across the out-of-bag observations. The increase of the prediction error has been computed for every tree, then averaged over the entire ensemble and divided by the standard deviation over the entire ensemble. This is the default approach of calculating feature importance when one sets the option “OOBVarImp” to “on” within TreeBagger in MATLAB. We computed the importance of each feature as we performed leave-one-out cross validation.

When one reduces the number of features used in the machine learning algorithm, the classifier becomes less complex and the time required for computation decreases. Therefore, we performed feature selection before building a final RF classifier used in the hidden test set. We built multiple RF classifiers with forward feature selection (incrementally adding the next most important feature).^28^ Then, we evaluated their performance using leave-one-out cross validation. We performed these procedures five times to assess the overall average performance as every RF classifier built is different, even if the same instances and features were input to the algorithm. From these five runs, we plotted the median and mean score curves vs. the number of features selected. We selected the number of features, *x*, at which the curves have roughly plateaued. To build our final RF classifiers for testing in the hidden dataset, we included 1.2*x (rounded up) number of features. In addition, we included the same features from both ECG signals in the final selection of features.

#### Prior

When the RF classifiers were built, the prior was set to uniform. This would make the classifier treat the majority and minority classes with equal importance instead of favoring the majority class in order to maximize the accuracy.

#### Cost-sensitive learning

The problem of optimal learning and decision-making with different misclassification errors incurring different penalties has been investigated before.^29^ As reflected in how the score is calculated in Eq. (3), misclassifying a true alarm as a false alarm has a more severe effect than misclassifying a false alarm as a true alarm. For purposes of this study, we fixed the cost of FP at 1 but varied the cost of FN and performed leave-one-out cross validation to find out the cost of FN that would maximize the score for each type of arrhythmia. Finally, in building the RF classifiers used for the hidden test set, the costs of FN that give the maximum score for each type of arrhythmia during the leave-one-out cross validation, were used.

#### RF classifiers built for the hidden test set

We built the final version of the RF classifiers using all data in the training set with feature selection and settings described above.

### Reporting summary

Further information on research design is available in the Nature Research Reporting Summary linked to this article.

## Supplementary information

Supplementary Information.

Reporting Summary.

## Data Availability

The training dataset will be available to any investigator upon request.

## Supplementary information

**Supplementary information** accompanies the paper on the *npj Digital Medicine* website (10.1038/s41746-019-0160-7).

## Code availability

The code will be available to any investigator upon request.

## References

[1] Drew BJ (2014). Insights into the problem of alarm fatigue with physiologic monitor devices: a comprehensive observational study of consecutive intensive care unit patients. PLoS ONE.

[2] Lawless ST (1994). Crying wolf: false alarms in a pediatric intensive care unit. Crit. Care Med..

[3] Donchin Y, Seagull FJ (2002). The hostile environment of the intensive care unit. Curr. Opin. Crit. Care.

[4] Balogh D, Kittinger E, Benzer A, Hackl J (1993). Noise in the ICU. Intensive Care Med..

[5] Keller JP (2012). Clinical alarm hazards: a “top ten” health technology safety concern. J. Electrocardiol..

[6] Cvach M (2012). Monitor alarm fatigue: an integrative review. Biomed. Instrum. Technol..

[7] Feldman JM, Ebrahim MH, Bar-Kana I (1997). Robust sensor fusion improves heart rate estimation: clinical evaluation. J. Clin. Monit..

[8] Mäkivirta A, Koski E, Kari A, Sukuvaara T (1991). The median filter as a preprocessor for a patient monitor limit alarm system in intensive care. Comput. Methods Prog. Biomed..

[9] Schmid F, Goepfert MS, Reuter DA (2013). Patient monitoring alarms in the ICU and in the operating room. Crit. Care.

[10] Aboukhalil A, Nielsen L, Saeed M, Mark RG, Clifford GD (2008). Reducing false alarm rates for critical arrhythmias using the arterial blood pressure waveform. J. Biomed. Inform..

[11] Li Q, Clifford GD (2012). Signal quality and data fusion for false alarm reduction in the intensive care unit. J. Electrocardiol..

[12] Imhoff M, Kuhls S (2006). Alarm algorithms in critical care monitoring. Anesth. Analg..

[13] Clifford GD (2015). The physionet/computing in cardiology challenge 2015: reducing false arrhythmia alarms in the ICU. Comput. Cardiol..

[14] Eerikainen LM, Vanschoren J, Rooijakkers MJ, Vullings R, Aarts RM (2016). Reduction of false arrhythmia alarms using signal selection and machine learning. Physiol. Meas..

[15] Kalidas V, Tamil LS (2016). Cardiac arrhythmia classification using multi-modal signal analysis. Physiol. Meas..

[16] Plesinger F, Klimes P, Halamek J, Jurak P (2016). Taming of the monitors: reducing false alarms in intensive care units. Physiol. Meas..

[17] Antink, C. H. & Leonhardt, S. in *Computing in Cardiology Conference (CinC)* 285–288 (IEEE, 2015).

[18] Johnson AE, Behar J, Andreotti F, Clifford GD, Oster J (2015). Multimodal heart beat detection using signal quality indices. Physiol. Meas..

[19] Bach TA, Berglund L-M, Turk E (2018). Managing alarm systems for quality and safety in the hospital setting. BMJ Open Qual..

[20] Zong, W., Heldt, T., Moody, G. & Mark, R. in *Computers in Cardiology* 259–262 (IEEE, 2003).

[21] Martinez JP, Almeida R, Olmos S, Rocha AP, Laguna P (2004). A wavelet-based ECG delineator: evaluation on standard databases. IEEE Trans. Bio-Med. Eng..

[22] Sun, J., Reisner, A. & Mark, R. in *Computers in Cardiology* 13–16 (IEEE, 2006).

[23] Breiman L (2001). Random forests. Mach. Learn..

[24] Sieben, W. & Gather, U. in *Conference on Artificial Intelligence in Medicine in Europe*. 130–138 (Springer, 2007).

[25] Quinlan JR (1986). Induction of decision trees. Mach. Learn..

[26] Lemon SC, Roy J, Clark MA, Friedmann PD, Rakowski W (2003). Classification and regression tree analysis in public health: methodological review and comparison with logistic regression. Ann. Behav. Med..

[27] Refaeilzadeh, P., Tang, L. & Liu, H. in *Encyclopedia of Database Systems* 532–538 (Springer, 2009).

[28] Guyon I, Elisseeff A (2003). An introduction to variable and feature selection. J. Mach. Learn. Res..

[29] Elkan, C. in *International Joint Conference on Artificial Intelligence* 973–978 (Lawrence Erlbaum Associates Ltd., 2001).

