# Supplementary material for: Reduction of false alarms in the intensive care unit using an optimized machine learning based approach
Source: NPJ Digit Med. 2019 Sep 5;2:86. doi: 10.1038/s41746-019-0160-7 (PMC6728371; doi:10.1038/s41746-019-0160-7)
Supplement: Supplementary file 1 — Supplementary Information. [file 41746_2019_160_MOESM1_ESM.pdf]

Reduction of False Alarms in the Intensive Care Unit Using an Optimized Machine Learning Based Approach

Supplementary Information

Wan-Tai M Au-Yeung, Ashish K Sahani, Eric M. Isselbacher, Antonis A. Armoundas

Supplementary Methods

Description of Features

Electrocardiogram (ECG) Features

Time-Domain ECG Features

Heart Rate

Heart rate is determined from the R peak detection. For tachycardia, the fastest average heart rate from a sequence of 16 consecutive heart beats is extracted from the 285th second to the 300th second of the records. For bradycardia, the slowest average heat rate from a sequence of 4 consecutive heart beats is extracted from the 285th second to the 300th second of the records.

Maximum Gap between R peaks

For asystole, the maximum time duration between consecutive R peaks in ECG signals was calculated and used as a feature.

Periodicity Measure (PM)

ECG signals generally follow a periodic rhythm in normal cases and in most arrhythmic cases except asystole and ventricular fibrillation (VF). One of the strongest markers of good signal quality for ECG is the degree of periodicity. Once R peaks are identified we obtain all peak-to-peak time periods and put them in an array $I = [I_1, I_2, \dots I_n]$. For highly periodic signals the standard deviation would be small for this array of time periods. We calculate the periodicity measure by equation (1):

$$PM = 100 - 100 * s_I/\bar{I} \qquad (1)$$

where s_I is the standard deviation of the array I and $\bar{I}$ is the mean value of the array I. PM is close to zero for highly aperiodic signal and close to 100 for highly periodic signals. Supplementary Figure 27 shows an illustration of the periodicity measure.

Sharpness Measure (SM)

A good quality ECG has sharp QRS complexes except in the cases of VF and VT. We quantify the sharpness, S_i, of the ith QRS complex by measuring the minimum absolute slope around the QRS complex. We calculate the sharpness for each QRS within the window of analysis and put it in an array $S = [S_1, S_2, \dots, S_n]$. The sharpness measure for an ECG signal within a window of analysis is given by equation (2):

$$SM = \left(\frac{200}{\pi}\right) * \tan^{-1}(\bar{S}) \qquad (2)$$

where $\bar{S}$ is the mean of the array S. SM can take values between 0 and 100. An ECG signal with highly sharp QRS complexes has values close to 100. Supplementary Figure 28 shows an illustration of the sharpness measure.

Correlation Measure (CM)

As a QRS complex is a repeating pattern in the ECG, it generally has a high beat-to-beat correlation. We calculate the correlation between n successive QRS complex detection and store them in an array $C = [C_1, C_2, \dots, C_{n-1}]$. Correlation measure is given by equation (3):

$$CM = 100 * \bar{C} \qquad (3)$$

where $\bar{C}$ is the mean of the array C.

CM can take a value between 0 and 100. If two QRS complexes are identical then CM is 100 while CM is low for QRS complexes with different morphologies. Similar method has been used to measure the correlation among beats before.[1] Supplementary Figure 29 shows an illustration of the correlation measure.

Peak Height Stability Measure (PHSM)

Stable peak heights would often indicate high signal quality. Therefore, we invented the peak height stability measure. Each peak height is found by subtracting the amplitude of the ECG signal at the R peak detection by the mean amplitude of the ECG signal. All the peak heights within the window of analysis are stored in an array $\delta P = [\delta P_1, \delta P_2, \dots, \delta P_n]$. We find the PHSM by equation (4):

$$PHSM = 100 - 100 * s_{\delta P}/\overline{\delta P} \qquad (4)$$

where $s_{\delta P}$ is the standard deviation of δP and $\overline{\delta P}$ is the mean of the array δP. Supplementary Figure 30 shows an illustration of the peak height stability measure.

Complexity Measure

Complexity measure is derived from the viewpoint of dynamical systems.[2] The complexity measure was calculated by comparison and accumulation operations from a string of zeros and ones, which is a reconstruction of the original ECG data for a specific window length and an appropriate threshold. This complexity measure has been shown to effectively detect sinus rhythm, VT and VF.

Not Enough Beats

We created this binary feature to indicate whether there are enough heartbeats within the window of analysis for calculation of heart rate for classifying tachycardia alarms. The number of heartbeats required for calculation of heart rate for tachycardia is 16.

Median Neighborhood Swing

For true and false alarms of asystole, neighborhood swing for each detected peak is calculated by first taking a sub segment of the ECG signal centered on the detected peak, one third of a second before and one third of a second after the detected peak. The swing is equal to the maximum of absolute value of the ECG sub segment minus the absolute value of the mean of the ECG sub segment. The median neighborhood swing is found by taking the median value of all neighborhood swing values within the window of analysis.

Blank Area Swing

For true and false alarms of asystole, if the maximum RR interval between consecutive R peaks is greater than or equal to 3.5s, the blank area swing would be calculated. Blank area is taken to start at the end of the first second of the maximum RR interval and end at the end of the third second of the maximum RR interval. Baseline wandering was first removed from the blank area using a 3rd order polynomial. Then, the blank area swing is equal to the maximum of absolute value of the blank area minus the absolute value of the mean of the blank area.

Blank Area Swing to Median Swing Ratio

For asystole, if the maximum RR interval is greater than or equal to 3.5s, the blank area swing to median swing ratio is also calculated and used as a feature.

Histogram normality

ECG spends most of the time in the baseline around zero. Thus, the histogram of ECG signal tends to have a Gaussian distribution centered on around zero with a small standard deviation. For bad-quality ECGs, VF and VT cases this condition is not satisfied and histogram can have very different mean and standard deviation. The mean and the standard deviation of the histogram were used as features for asystole alarms. Supplementary Figure 31 shows an example of a good ECG signal and an example of a bad ECG signal.

Meeting the criteria of VT

We created a binary feature that indicated whether the ECG signals met the criteria of VT. If a sequence of five consecutive VT beats was found and these VT beats all occurred within 2.4 seconds, then this binary feature would be given the value of one. Otherwise, this binary feature would be given the value of zero.

Low Frequency Power Dominant

After applying the method of amplitude envelope, one can conclude which frequency power band is the most dominant at a certain point in time. During VF, the low frequency power band should be the most dominant as VF resembles a signal of frequency 2.5-8Hz.[3] Therefore, we invented a binary feature, Low Frequency Power Dominant, to indicate whether low frequency power was dominant for 4 seconds continuously for VF alarms.

Frequency-Domain ECG Features

For detection of certain arrhythmia such as VF and VT, it is worthwhile to analyze ECGs in the frequency domain. We performed Fast Fourier Transform (FFT) of ECGs in records that caused VT and VF alarms and then extracted features from the frequency spectrums. Supplementary Figure 32 shows the frequency spectrum of a true VF event. Features extracted include the following:

Dominant Frequency

Dominant frequency is the frequency at which the power spectrum has its highest power.[3] For VF, the dominant frequency should be in the range of 2.5-8Hz.

Maximum power to total power ratio

We hypothesized that during VF the ECG would have most of its power in a single frequency. Therefore, we invented this feature which is the maximum power to total power ratio in the frequency domain to examine how concentrated the power is in a single frequency. ECGs during VF would have a higher maximum power to total power ratio than normal ECGs.

Co-dominant frequencies

This refers to the number of significant frequency components besides the dominant frequency. These frequency components have minimum peak heights of 0.2 in the normalized power spectrum.[4] This is another measure that describe how concentrated the power is at the dominant frequency.

Bandwidth

Here we define the bandwidth of the ECG signal as the difference between the last and first frequencies in the normalized power spectrum that exceeds power of 0.5. During VF, the bandwidth of the ECG signal would decrease significantly.

Mean frequency

The frequency spectrum is characterized by its mean frequency which is the sum of the product of the spectrum intensity and its respective frequency, divided by the total sum of spectrum intensity. This is shown in equation (5):

$$f_{mean} = \frac{\sum I * f}{\sum I} \qquad (5)$$

where f is the frequency and I is the spectrum intensity.

Median frequency

The frequency spectrum is also characterized by its median frequency. To find the median frequency, one has to calculate the total power of the whole spectrum first. Then, the median frequency is the frequency at which the cumulative power (sum of all the power for lower frequencies) first exceeds half of the total power.[5]

Blood Pressure (BP) and Photoplethysmogram (PPG) Features

Features for Both BP and PPG

Heart Rate Estimated from the Onsets of Waveforms

Besides using the ECG signals, heart rates were also estimated from the onsets of waveforms in the BP and PPG signals separately.

Periodicity Measure

The periods between the onsets of n waveforms were calculated and stored in an array $I = [I_1, I_2, \ldots, I_{n-1}]$. For highly periodic signals the standard deviation would be small for this array of time periods. The equation used for calculating periodicity measure for BP or PPG is the same as that for ECG as illustrated in equation (1). Supplementary Figure 33 shows an illustration of this periodicity measure.

Correlation Measure

High-quality BP and PPG signals are often very regular. Therefore, we calculated the cross-correlation coefficients between n consecutive waveforms and put them in an array $C = [C_1, C_2, \ldots, C_{n-1}]$. The correlation measure for the BP or PPG signals within a window of analysis is the mean of these cross-correlation coefficients multiplied by a hundred. This is described in equation (3). Supplementary Figure 34 shows an illustration of this correlation measure.

δP Stability Measure

When the signal quality is high, the value of the maximum amplitude minus the minimum amplitude for each waveform would be quite stable during sinus rhythm in BP and PPG signals. Such values were calculated for all detected waveforms within the window of analysis and stored in an array $\delta P = [\delta P_1, \delta P_2, \ldots, \delta P_n]$. δP Stability Measure was calculated using equation (4). Supplementary Figure 35 shows an illustration of this δP Stability Measure.

Decreasing δP

During VT, BP and PPG amplitude would often gradually decrease. Therefore, the binary feature, decreasing δP, was invented to indicate whether the amplitude of the BP signal or PPG signal keep on decreasing.

Absence of Peaks

During VF, there should be no onsets of waveforms in BP and PPG signals because the heart is not pumping blood. Therefore, we created two binary features, absence of peaks, one for the BP signal and another one for the PPG signal, to indicate whether there are onsets of waveforms in the BP and PPG signals for VF alarms.

Maximum Gap between Consecutive Onsets

For asystole alarms, we calculated the maximum gaps between consecutive onsets of waveforms in BP and PPG signals respectively and used them as features.

BP-only Features

Maximum Blood Pressure before Onset of Largest Gap

Maximum BP before the occurrence of largest gap between consecutive onsets of waveforms is used as a feature.

Maximum Blood Pressure during the Last Second of Largest Gap

When the heart does not beat for a long time, the blood pressure would decrease. The maximum blood pressure during the last second of the largest gap between consecutive onsets of waveforms in BP is used as a feature.

Blood Pressure Decrease

This is a binary feature that indicate whether the maximum blood pressure during the last second of largest gap is lower than the maximum blood pressure before the onset of largest gap.

PPG-only Features

PPG Maximum Amplitude before Onset

This is the largest amplitude of PPG before the onset of the largest gap between consecutive valleys in the PPG.

PPG Maximum Amplitude after Onset

This is the largest amplitude of PPG within the largest gap between consecutive valleys in the PPG.

PPG Amplitude Decrease

This is a binary feature that indicates whether the PPG amplitude decreases after the onset of the largest gap.

Supplementary References

1. Orphanidou, C. *et al.* Signal-quality indices for the electrocardiogram and photoplethysmogram: derivation and applications to wireless monitoring. *IEEE journal of biomedical and health informatics* **19**, 832-838, doi:10.1109/JBHI.2014.2338351 (2015).
2. Zhang, X. S., Zhu, Y. S., Thakor, N. V. & Wang, Z. Z. Detecting ventricular tachycardia and fibrillation by complexity measure. *IEEE transactions on bio-medical engineering* **46**, 548-555 (1999).
3. Plesinger, F., Klimes, P., Halamek, J. & Jurak, P. Taming of the monitors: reducing false alarms in intensive care units. *Physiol Meas* **37**, 1313-1325, doi:10.1088/0967-3334/37/8/1313 (2016).
4. Kalidas, V. & Tamil, L. S. Cardiac arrhythmia classification using multi-modal signal analysis. *Physiol Meas* **37**, 1253-1272, doi:10.1088/0967-3334/37/8/1253 (2016).
5. Dzwonczyk, R., Brown, C. G. & Werman, H. A. The median frequency of the ECG during ventricular fibrillation: its use in an algorithm for estimating the duration of cardiac arrest. *IEEE transactions on bio-medical engineering* **37**, 640-646, doi:10.1109/10.55668 (1990).

Supplementary Table 1: Cost of FN that maximize the score in the leave-one-out cross validation for each type of arrhythmia

	Cost of FN
Asystole	1
Bradycardia	1
Tachycardia	1.4
Ventricular Fibrillation	1.2
Ventricular Tachycardia	1.2

Supplementary Table 2: List of Features Considered and List of Features Selected for Building the Random Forest for Asystole

List of Features Considered	List of Features Selected Arranged With Their Importance in Descending Order
1. ECG 1 periodicity measure	ECG 1 maximum gap between consecutive R peaks
2. ECG 1 sharpness measure	ECG 2 blank area swing to median swing ratio
3. ECG 1 correlation measure	ECG 1 blank area swing
4. ECG 1 peak height stability measure	ECG 2 maximum gap between consecutive R peaks
5. ECG 1 maximum gap between consecutive R peaks	ECG 1 median swing
6. ECG 1 histogram analysis mean	ECG 2 blank area swing
7. ECG 1 histogram analysis standard deviation	ECG 2 histogram analysis standard deviation
8. ECG 1 median swing	ECG 1 sharpness measure
9. ECG 1 blank area swing	ECG 2 correlation measure
10. ECG 1 blank area swing to median swing ratio	PPG periodicity measure
11. ECG 2 periodicity measure	ECG 1 blank area swing to median swing ratio
12. ECG 2 sharpness measure	ECG 1 histogram analysis standard deviation
13. ECG 2 correlation measure	PPG maximum gap between consecutive valleys
14. ECG 2 peak height stability measure	PPG correlation measure
15. ECG 2 maximum gap between consecutive R peaks	ECG 1 histogram analysis mean
16. ECG 2 histogram analysis mean	ECG 2 sharpness measure
17. ECG 2 histogram analysis standard deviation	ECG 1 periodicity measure
18. ECG 2 median swing	ECG 2 histogram analysis mean
19. ECG 2 blank area swing	ECG 1 correlation measure
20. ECG 2 blank area swing to median swing ratio	ECG 2 peak height stability measure
21. BP periodicity measure	ECG 2 periodicity measure
22. BP δP stability measure	ECG 2 median swing
23. BP correlation measure	BP maximum gap between consecutive valleys
24. BP maximum gap between consecutive valleys	PPG δP stability measure
25. BP minimum pressure at maximum gap	ECG 1 peak height stability measure
26. BP pressure decrease	
27. BP maximum pressure before onset	
28. BP maximum pressure after onset	
29. PPG periodicity measure	
30. PPG δP stability measure	
31. PPG correlation measure	
32. PPG maximum gap between consecutive valleys	
33. PPG amplitude decrease	
34. PPG maximum amplitude before onset	
35. PPG maximum amplitude after onset	

Supplementary Table 3: List of Features Considered and List of Features Selected for Building the Random Forest for Bradycardia

List of Features Considered	List of Features Selected Arranged With Their Importance in Descending Order
1. ECG 1 periodicity measure<br>2. ECG 1 sharpness measure<br>3. ECG 1 correlation measure<br>4. ECG 1 peak height stability measure<br>5. ECG 1 minimum heart rate<br>6. ECG 1 number of beats slower than 46bpm<br>7. ECG 2 periodicity measure<br>8. ECG 2 sharpness measure<br>9. ECG 2 correlation Measure<br>10. ECG 2 peak height stability measure<br>11. ECG 2 minimum heart rate<br>12. ECG 2 number of beats slower than 46bpm<br>13. BP periodicity measure<br>14. BP δP stability measure<br>15. BP correlation measure<br>16. BP minimum heart rate<br>17. BP number of beats slower than 46bpm<br>18. PPG periodicity measure<br>19. PPG δP stability measure<br>20. PPG correlation measure<br>21. PPG minimum heart rate<br>22. PPG number of beats slower than 46bpm	ECG 2 minimum heart rate<br>ECG 1 minimum heart rate<br>PPG minimum heart rate<br>ECG 2 number of beats slower than 46bpm<br>BP number of beats slower than 46bpm<br>BP minimum heart rate<br>PPG periodicity measure<br>ECG 2 correlation measure<br>PPG number of beats slower than 46bpm<br>ECG 1 number of beats slower than 46bpm<br>ECG 2 peak height stability measure<br>PPG δP stability measure<br>BP periodicity measure<br>ECG 1 peak height stability measure<br>ECG 2 sharpness measure<br>PPG correlation measure<br>ECG 1 sharpness measure<br>ECG 1 correlation measure

Supplementary Table 4: List of Features Considered and List of Features Selected for Building the Random Forest for Tachycardia

List of Features Considered	List of Features Selected Arranged With Their Importance in Descending Order
1. ECG 1 periodicity measure<br>2. ECG 1 sharpness measure<br>3. ECG 1 correlation measure<br>4. ECG 1 peak height stability measure<br>5. ECG 1 maximum heart rate<br>6. ECG 1 not enough beats<br>7. ECG 2 periodicity measure<br>8. ECG 2 sharpness measure<br>9. ECG 2 correlation Measure<br>10. ECG 2 peak height stability measure<br>11. ECG 2 maximum heart rate<br>12. ECG 2 not enough beats<br>13. BP periodicity measure<br>14. BP δP stability measure<br>15. BP correlation measure<br>16. BP maximum heart rate<br>17. BP not enough beats<br>18. PPG periodicity measure<br>19. PPG δP stability measure<br>20. PPG correlation measure<br>21. PPG maximum heart rate<br>22. PPG not enough beats	ECG 2 maximum heart rate<br>ECG 1 maximum heart rate<br>ECG 1 correlation measure<br>ECG 1 peak height stability measure<br>ECG 2 sharpness measure<br>BP periodicity measure<br>ECG 2 peak height stability measure<br>PPG periodicity measure<br>ECG 2 periodicity measure<br>ECG 1 sharpness measure<br>ECG 2 correlation measure<br>BP correlation measure<br>BP δP stability measure<br>ECG 1 periodicity measure<br>PPG δP stability measure

Supplementary Table 5: List of Features Considered and List of Features Selected for Building the Random Forest for VF

List of Features Considered	List of Features Selected Arranged With Their Importance in Descending Order
1. ECG 1 periodicity measure	ECG 1 mean frequency
2. ECG 1 sharpness measure	ECG 1 median frequency
3. ECG 1 correlation measure	ECG 1 maximum power to total power ratio
4. ECG 1 peak height stability measure	Absence of BP peaks
5. ECG 1 dominant frequency	ECG 2 maximum power to total power ratio
6. ECG 1 bandwidth	PPG periodicity Measure
7. ECG 1 complexity	ECG 1 bandwidth
8. ECG 1 mean frequency	ECG 1 number of peaks with normalized power of at least 0.2
9. ECG 1 median frequency	PPG δP stability measure
10. ECG 1 maximum power to total power ratio	ECG 2 number of peaks with normalized power of at least 0.2
11. ECG 1 number of peaks with normalized power of at least 0.2	ECG 2 sharpness measure
12. ECG 1 LFP dominant	ECG 2 mean frequency
13. ECG 2 periodicity measure	ECG 2 bandwidth
14. ECG 2 sharpness measure	ECG 2 correlation measure
15. ECG 2 correlation measure	ECG 2 median frequency
16. ECG 2 peak height stability measure	BP periodicity measure
17. ECG 2 dominant frequency	ECG1 dominant frequency
18. ECG 2 bandwidth	ECG1 peak height stability measure
19. ECG 2 complexity	ECG2 dominant frequency
20. ECG 2 mean frequency	BP correlation measure
21. ECG 2 median frequency	PPG correlation measure
22. ECG 2 maximum power to total power ratio	ECG1 complexity
23. ECG 2 number of peaks with normalized power of at least 0.2	ECG2 periodicity measure
24. ECG 2 LFP dominant	ECG2 peak height stability measure
25. BP periodicity measure	ECG1 correlation measure
26. BP δP stability measure	ECG1 sharpness measure
27. BP correlation measure	BP δP stability measure
28. Absence of BP peaks	ECG2 complexity
29. PPG periodicity measure	ECG1 periodicity measure
30. PPG δP stability measure	
31. PPG correlation Measure	
32. Absence of PPG peaks	

Supplementary Table 6: List of Features Considered and List of Features Selected for Building the Random Forest for VT

List of Features Considered	List of Features Selected Arranged With Their Importance in Descending Order
1. ECG 1 meet VT criteria	ECG 2 correlation measure
2. ECG 1 periodicity measure	ECG 1 peak height stability measure
3. ECG 1 sharpness measure	ECG 2 peak height stability measure
4. ECG 1 correlation measure	ECG 2 periodicity measure
5. ECG 1 peak height stability measure	ECG 1 periodicity measure
6. ECG 1 complexity measure	ECG 1 meet VT criteria
7. ECG 1 minimum bandwidth	ECG 1 correlation measure
8. ECG 1 maximum power to total power ratio	ECG 1 complexity measure
9. ECG 1 number of peaks with normalized power of at least 0.2	ECG 1 minimum bandwidth
10. ECG 1 heart rate for segment	ECG 1 heart rate for segment
11. ECG 2 meet VT criteria	ECG 2 number of peaks with normalized power of at least 0.2
12. ECG 2 periodicity measure	ECG 2 sharpness measure
13. ECG 2 sharpness measure	ECG 1 number of peaks with normalized power of at least 0.2
14. ECG 2 correlation measure	ECG 1 sharpness measure
15. ECG 2 peak height stability measure	ECG 2 minimum bandwidth
16. ECG 2 complexity measure	ECG 1 maximum power to total power ratio
17. ECG 2 minimum bandwidth	ECG 2 heart rate for segment
18. ECG 2 maximum power to total power ratio	ECG 2 maximum power to total power ratio
19. ECG 2 number of peaks with normalized power of at least 0.2	ECG 2 complexity measure
20. ECG 2 heart rate for segment	PPG δP stability measure
21. BP periodicity measure	PPG correlation measure
22. BP δP stability measure	PPG periodicity measure
23. BP correlation measure	BP periodicity measure
24. BP decreasing δP	ECG 2 meet VT criteria
25. PPG periodicity measure	BP correlation measure
26. PPG δP stability measure	BP δP stability measure
27. PPG correlation measure	BP decreasing δP
28. PPG decreasing δP	PPG decreasing δP

Supplementary Table 7: Confusion Matrix

Outcome		Gold Standard	
		True Alarm	False Alarm
Predicted	True Alarm	True Positive (TP)	False Positive (FP)
	False Alarm	False Negative (FN)	True Negative (TN)

Supplementary Table 8: Number of seconds of each record analyzed for each type of arrhythmia

	Number of seconds of each record analyzed
Asystole	15
Bradycardia	15
Tachycardia	15
Ventricular Fibrillation	4
Ventricular Tachycardia	10

Supplementary Table 9: Scores from Leave-One-Out Cross Validation on the Training Set for Support Vector Machine, Neural Network and Random Forest for the Five Types of Arrhythmias

	Asystole	Bradycardia	Tachycardia	Ventricular Fibrillation	Ventricular Tachycardia
Support Vector Machine	72	48	98	68	45
Random Forest	85	59	95.8	73	61
Neural Network	76	54	90	68	53

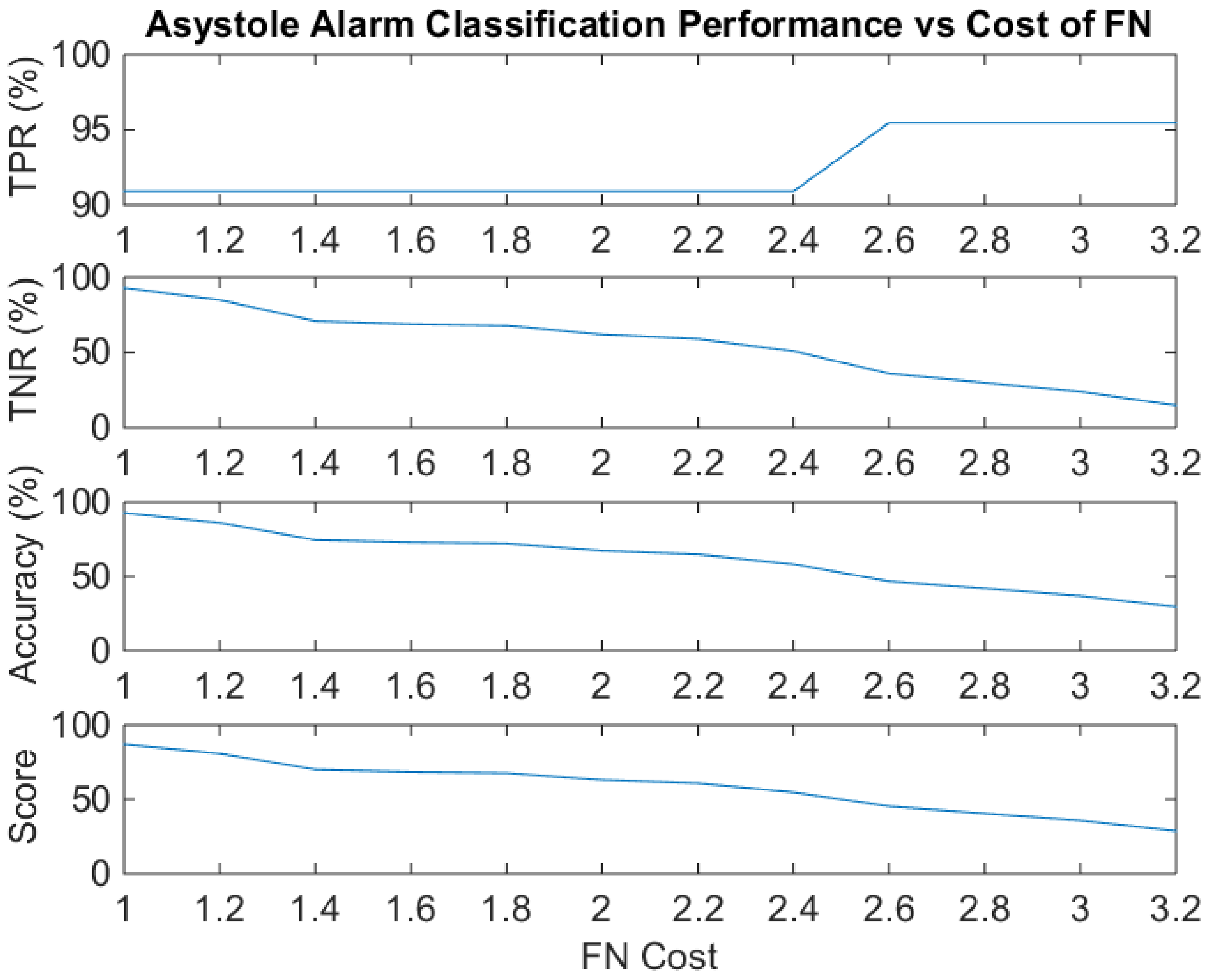

Supplementary Figure 1: Asystole – Performance vs FN Cost. Leave-one-out cross-validation asystole alarm classification performance vs cost of false negative (FN). The score is highest at cost of FN equals to 1.

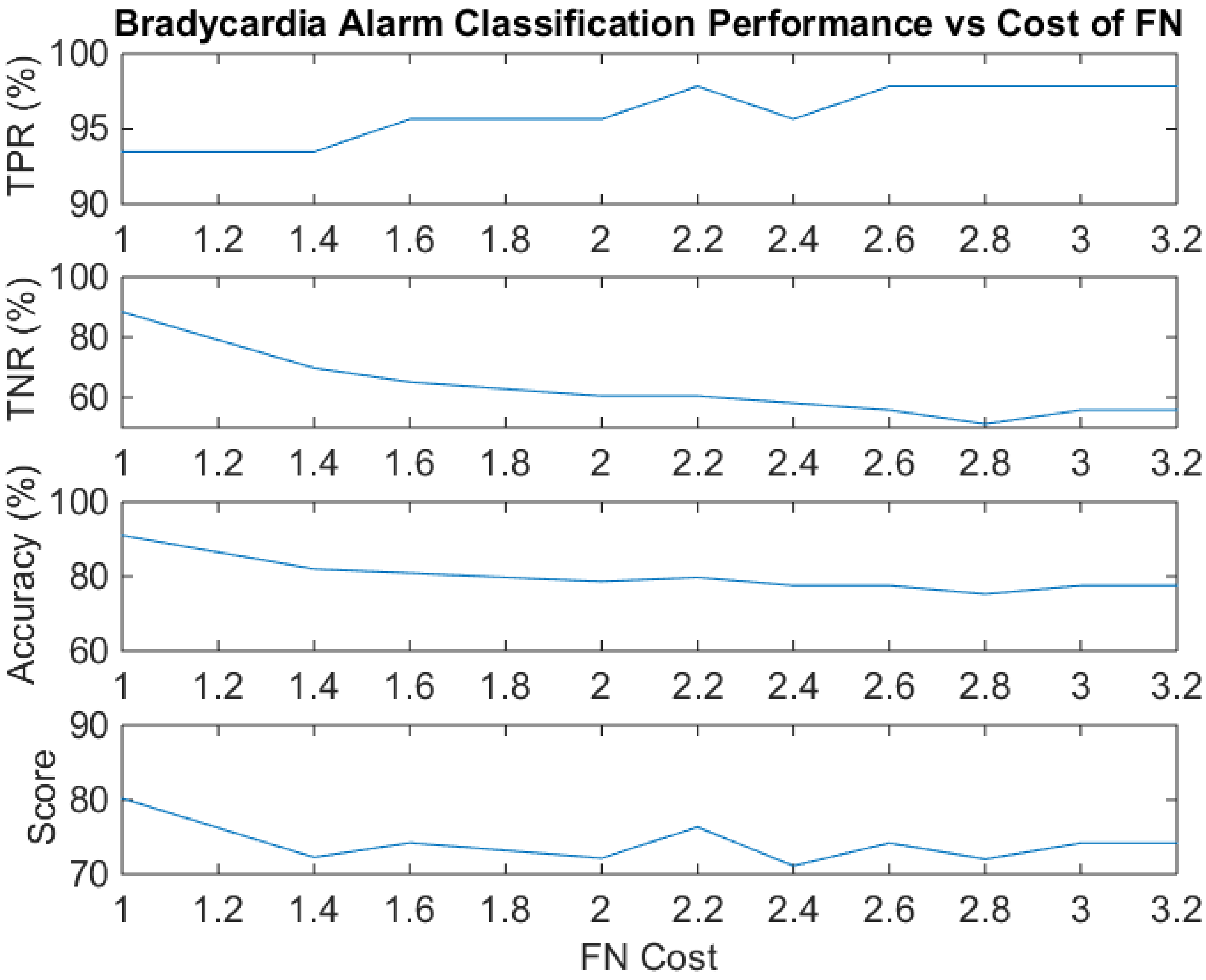

Supplementary Figure 2: Bradycardia – Performance vs FN Cost. Leave-one-out cross-validation bradycardia alarm classification performance vs cost of false negative (FN). The score is highest at cost of FN equals to 1.

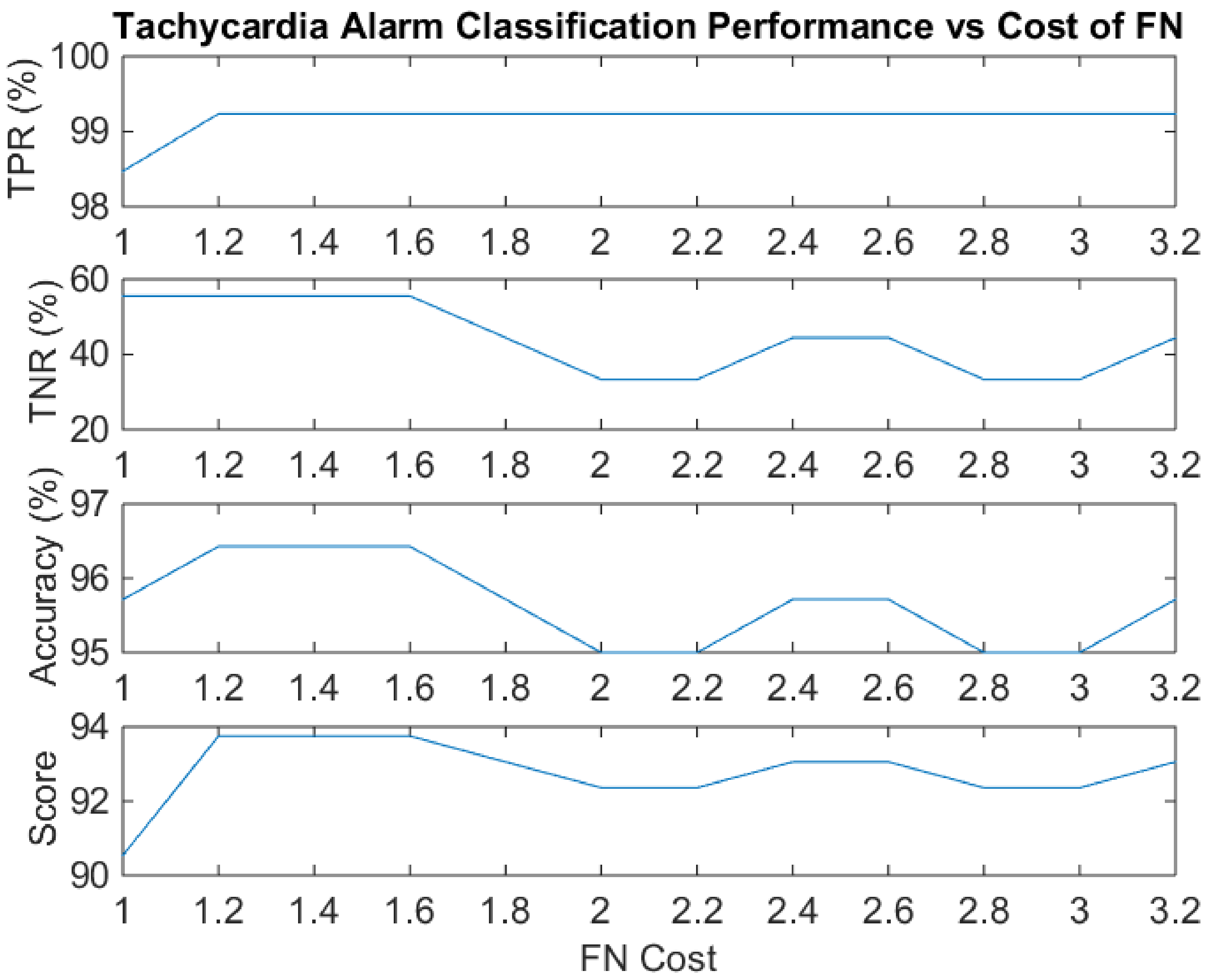

Supplementary Figure 3: Tachycardia – Performance vs FN Cost. Leave-one-out cross-validation tachycardia alarm classification performance vs cost of false negative (FN). The score is highest at cost of FN equals to 1.2, 1.4 and 1.6.

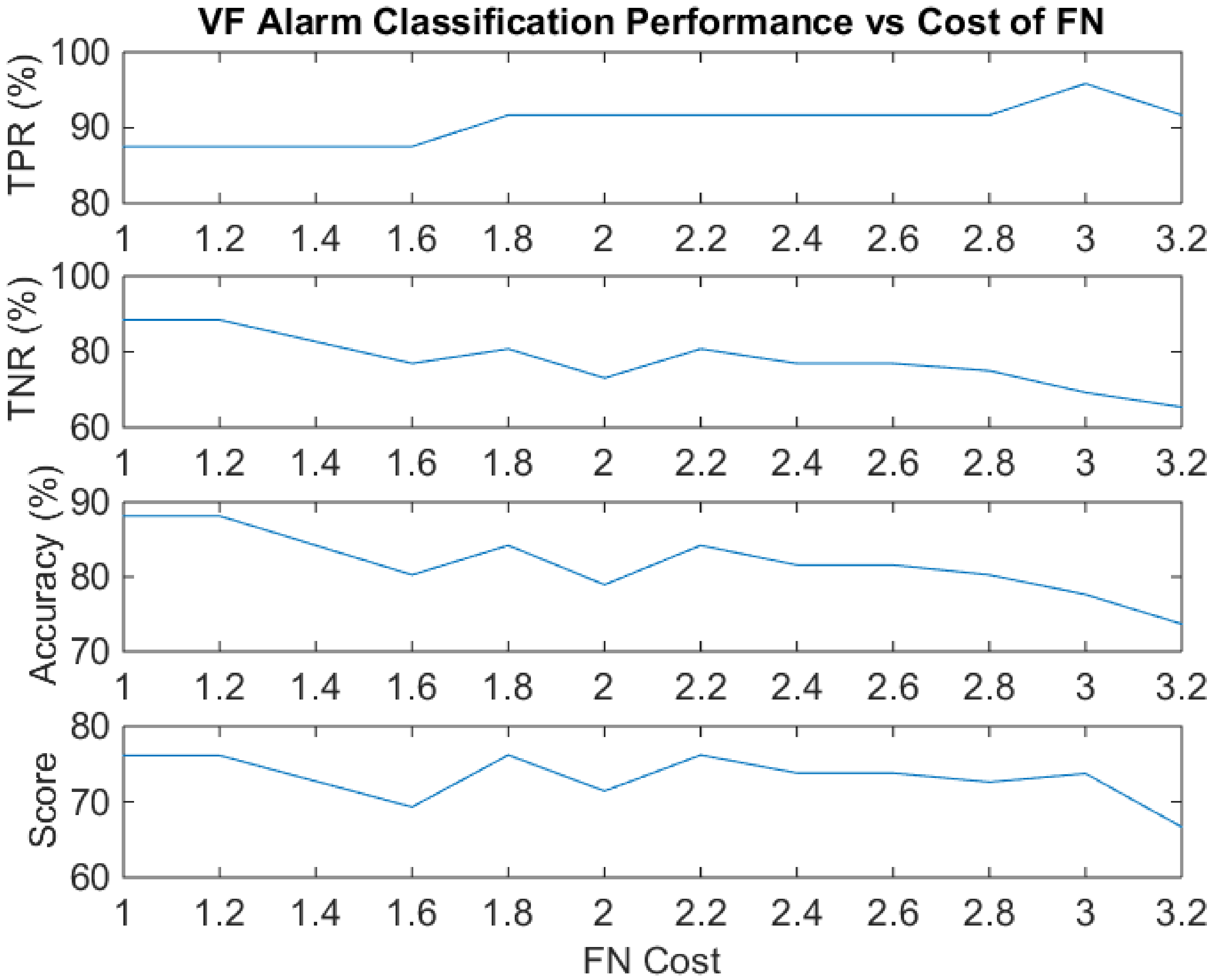

Supplementary Figure 4: VF – Performance vs FN Cost. Leave-one-out cross-validation VF alarm classification performance vs cost of false negative (FN). The score is highest at cost of FN equals to 1 and 1.2.

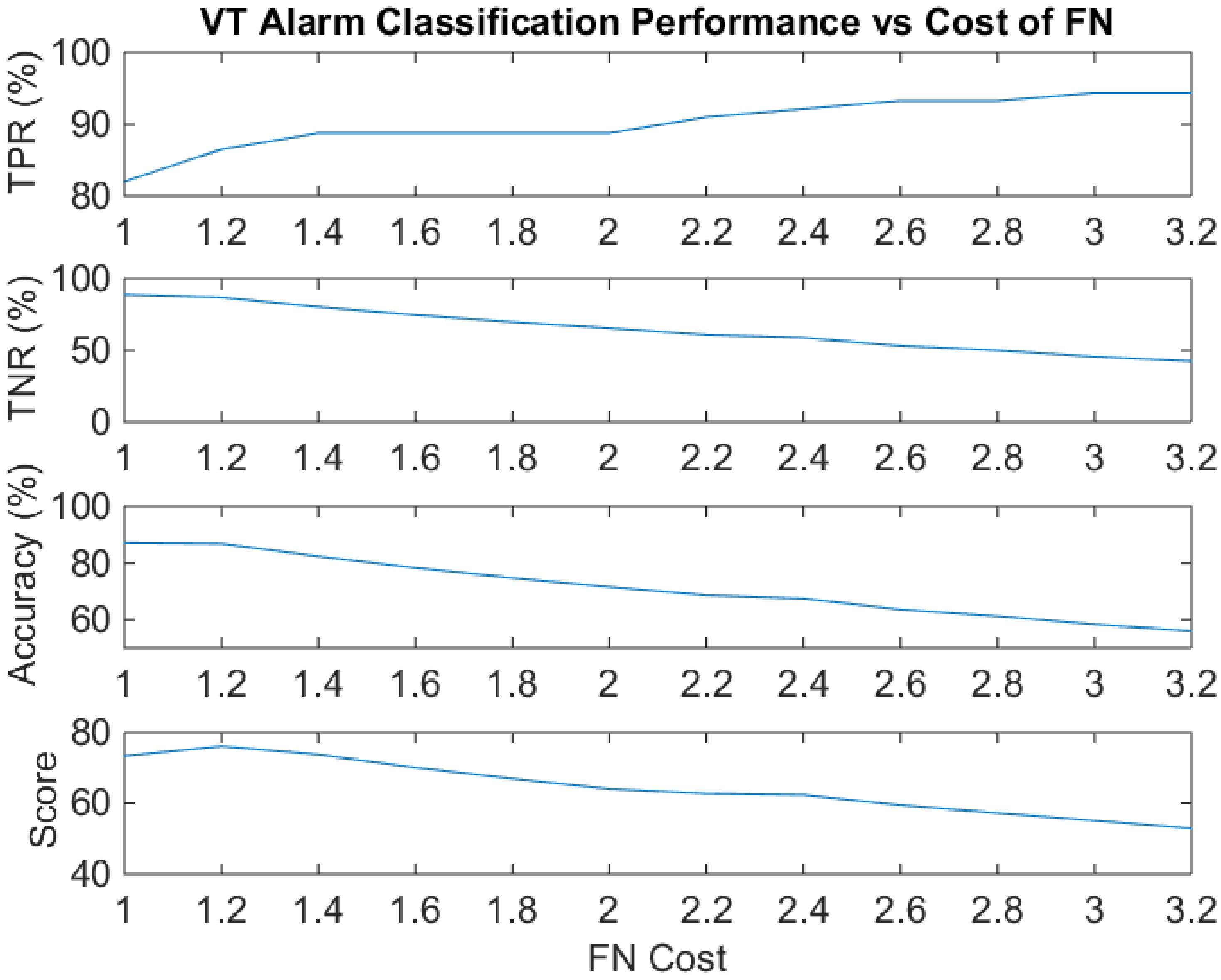

Supplementary Figure 5: VT – Performance vs FN Cost. Leave-one-out cross-validation VT alarm classification performance vs cost of false negative (FN). The score is highest at cost of FN equals to 1.2.

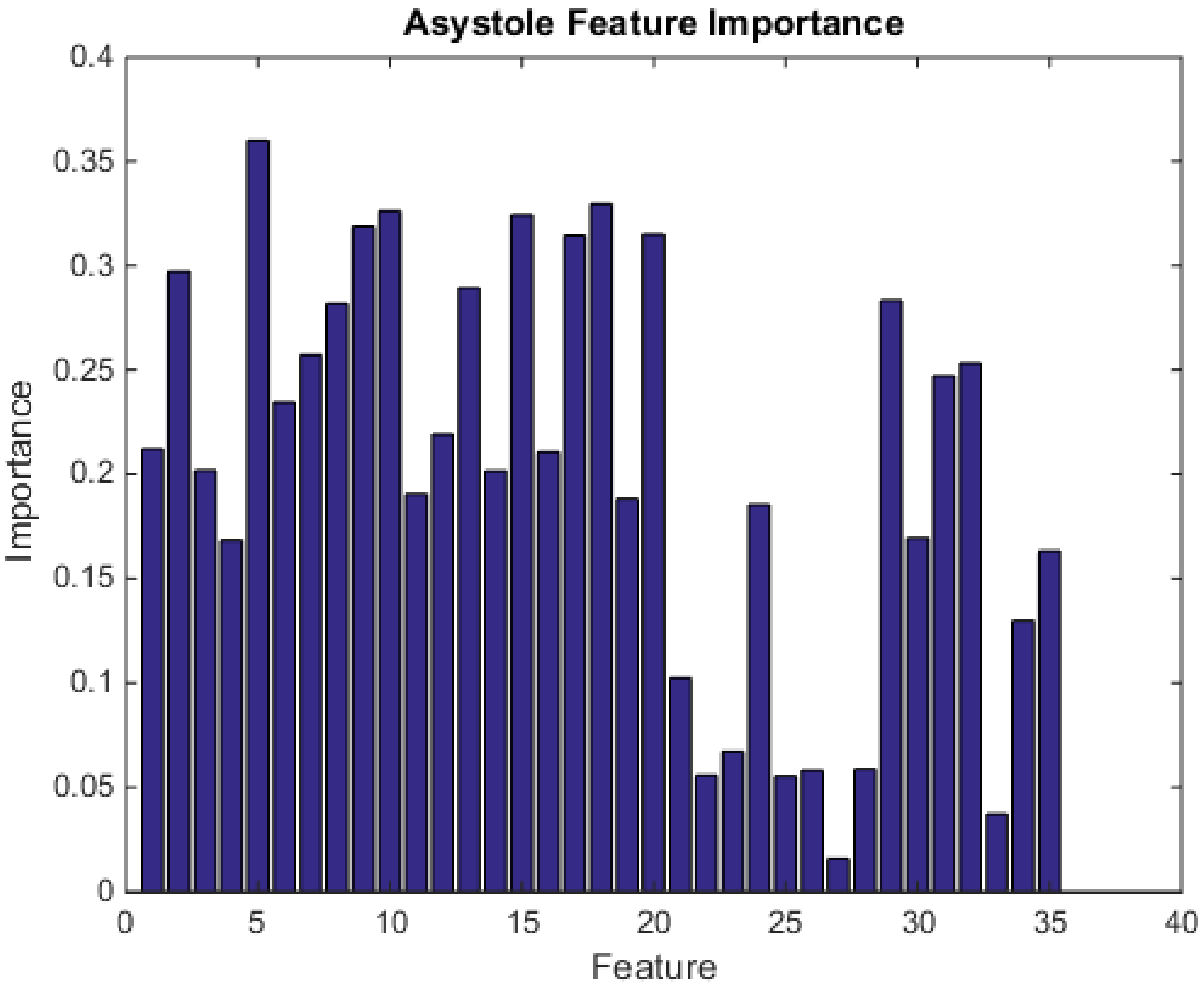

1. ECG 1 periodicity measure
2. ECG 1 sharpness measure
3. ECG 1 correlation measure
4. ECG 1 peak height stability measure
5. ECG 1 max gap between consecutive R waves
6. ECG 1 histogram analysis mean
7. ECG 1 histogram analysis standard deviation
8. ECG 1 median swing
9. ECG 1 blank area swing
10. ECG 1 blank area swing to median swing ratio
11. ECG 2 periodicity measure
12. ECG 2 sharpness measure
13. ECG 2 correlation measure
14. ECG 2 peak height stability measure
15. ECG 2 max gap between consecutive R waves
16. ECG 2 histogram analysis mean
17. ECG 2 histogram analysis standard deviation
18. ECG 2 median swing
19. ECG 2 blank area swing
20. ECG 2 blank area swing to median swing ratio
21. BP periodicity measure
22. BP δP stability measure
23. BP correlation measure
24. BP max gap between consecutive valleys
25. BP min pressure at max gap
26. BP pressure decrease
27. BP max pressure before onset
28. BP max pressure after onset
29. PPG periodicity measure
30. PPG δP stability measure
31. PPG correlation measure
32. PPG max gap between consecutive valleys
33. PPG amplitude decrease
34. PPG max amplitude before onset
35. PPG max amplitude after onset

Supplementary Figure 6: Asystole – Importance of Features. Importance of features for classification of asystole alarms calculated with the random forest algorithms.

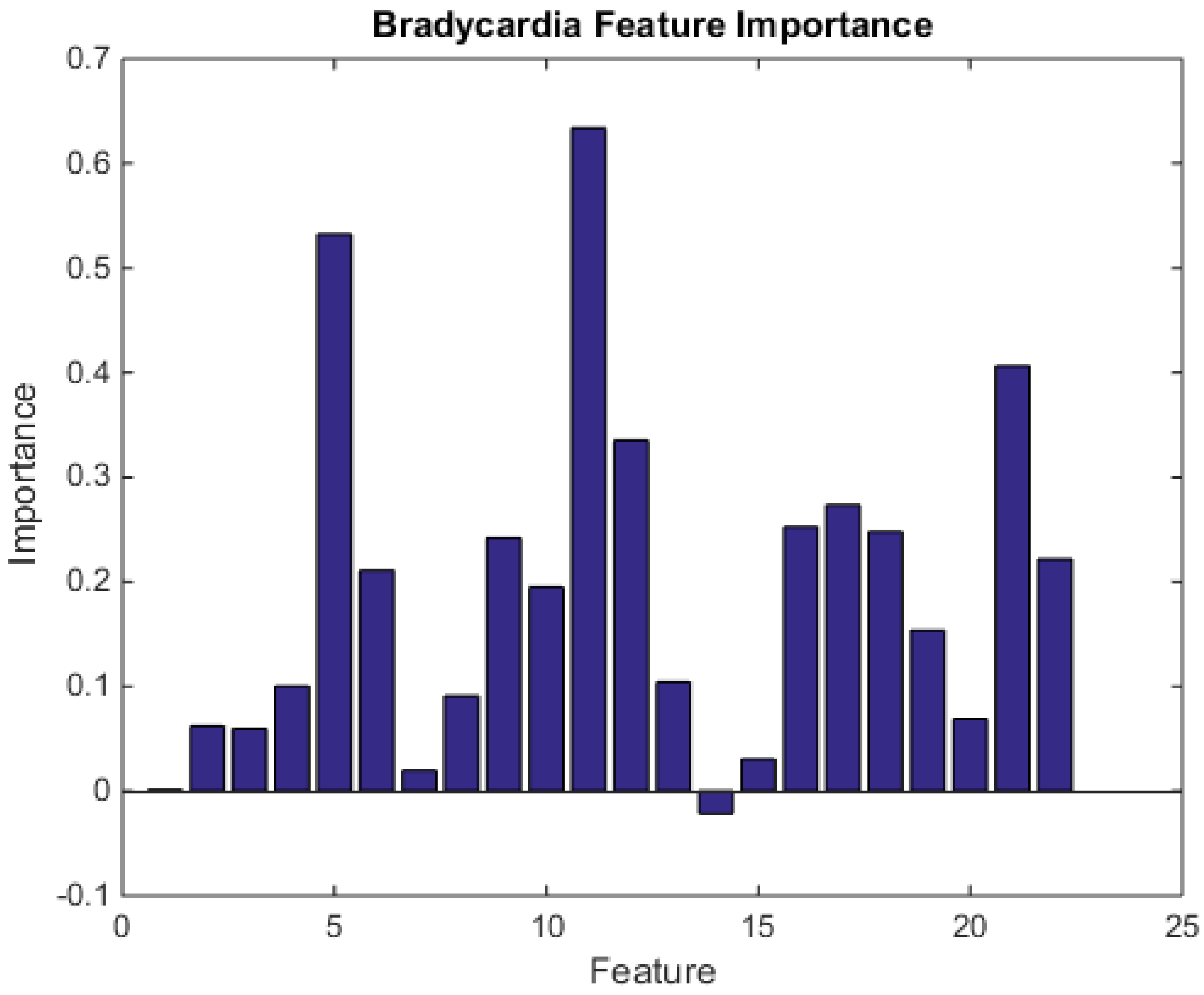

1. ECG 1 periodicity measure
2. ECG 1 sharpness measure
3. ECG 1 correlation measure
4. ECG 1 peak height stability measure
5. ECG 1 minimum heart rate
6. ECG 1 number of beats slower than 46bpm
7. ECG 2 periodicity measure
8. ECG 2 sharpness measure
9. ECG 2 correlation Measure
10. ECG 2 peak height stability measure
11. ECG 2 minimum heart rate
12. ECG 2 number of beats slower than 46bpm
13. BP periodicity measure
14. BP δP stability measure
15. BP correlation measure
16. BP minimum heart rate
17. BP number of beats slower than 46bpm
18. PPG periodicity measure
19. PPG δP stability measure
20. PPG correlation measure
21. PPG minimum heart rate
22. PPG number of beats slower than 46bpm

Supplementary Figure 7: Bradycardia – Importance of Features. Importance of features for classification of bradycardia alarms calculated with the random forest algorithms.

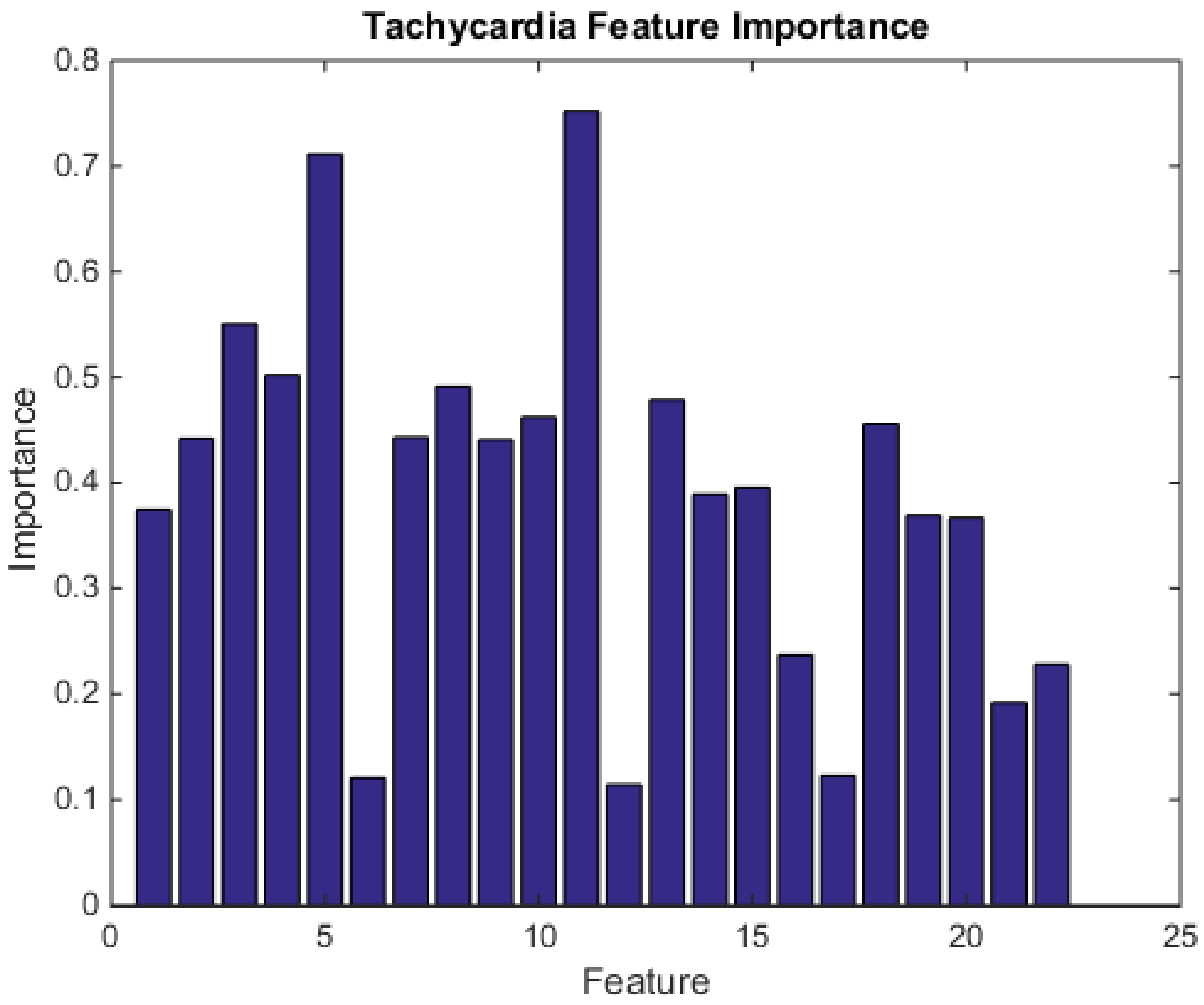

1. ECG 1 periodicity measure
2. ECG 1 sharpness measure
3. ECG 1 correlation measure
4. ECG 1 peak height stability measure
5. ECG 1 maximum heart rate
6. ECG 1 not enough beats
7. ECG 2 periodicity measure
8. ECG 2 sharpness measure
9. ECG 2 correlation Measure
10. ECG 2 peak height stability measure
11. ECG 2 maximum heart rate
12. ECG 2 not enough beats
13. BP periodicity measure
14. BP δP stability measure
15. BP correlation measure
16. BP maximum heart rate
17. BP not enough beats
18. PPG periodicity measure
19. PPG δP stability measure
20. PPG correlation measure
21. PPG maximum heart rate
22. PPG not enough beats

Supplementary Figure 8: Tachycardia – Importance of Features. Importance of features for classification of tachycardia alarms calculated with the random forest algorithms.

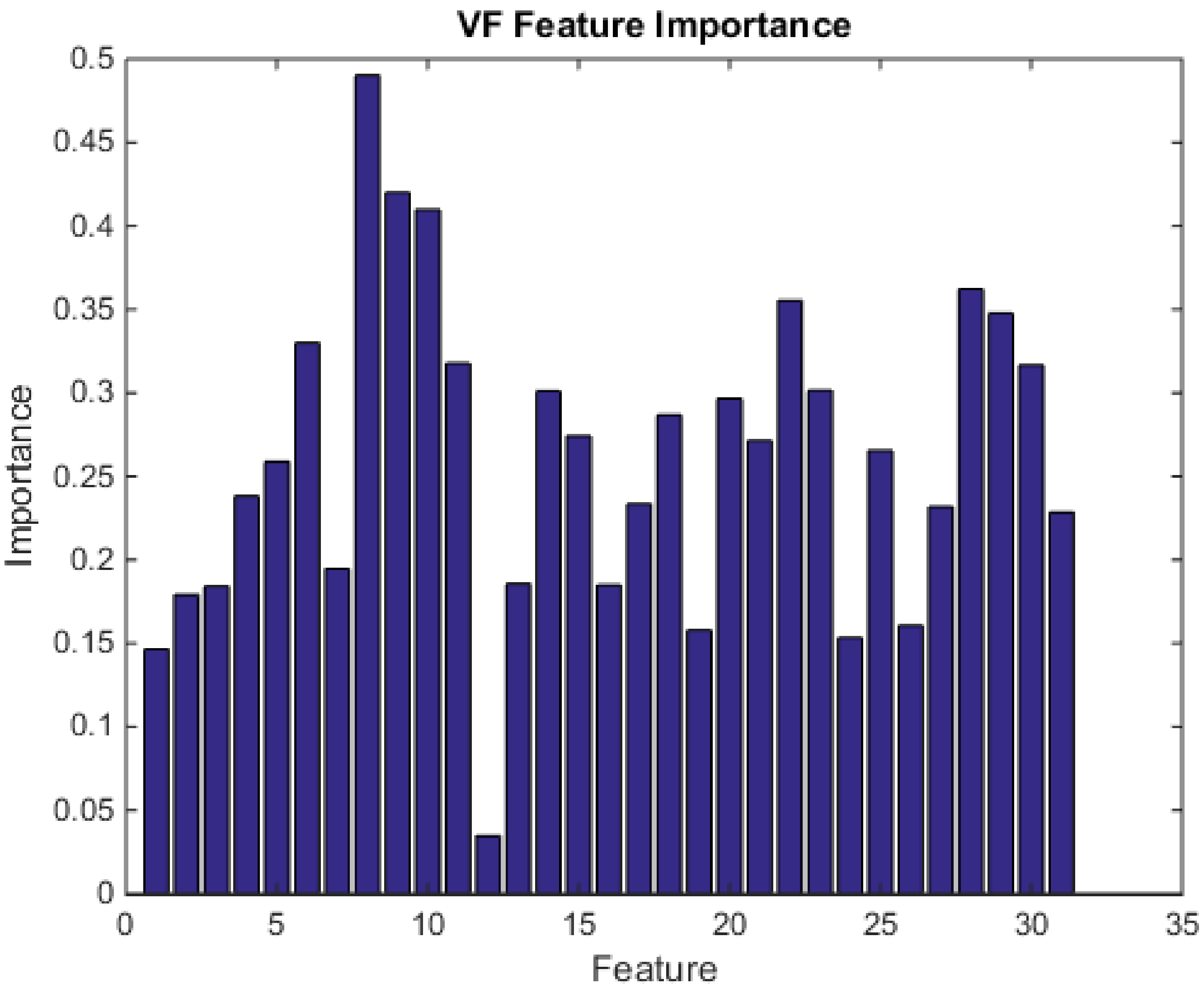

1. ECG 1 periodicity measure
2. ECG 1 sharpness measure
3. ECG 1 correlation measure
4. ECG 1 peak height stability measure
5. ECG 1 dominant frequency
6. ECG 1 bandwidth
7. ECG 1 complexity
8. ECG 1 mean frequency
9. ECG 1 median frequency
10. ECG 1 max power to total power ratio
11. ECG 1 number of peaks with normalized power of at least 0.2
12. ECG 1 LFP dominant
13. ECG 2 periodicity measure
14. ECG 2 sharpness measure
15. ECG 2 correlation measure
16. ECG 2 peak height stability measure
17. ECG 2 dominant frequency
18. ECG 2 bandwidth
19. ECG 2 complexity
20. ECG 2 mean frequency
21. ECG 2 median frequency
22. ECG 2 max power to total power ratio
23. ECG 2 number of peaks with normalized power of at least 0.2
24. ECG 2 LFP dominant
25. BP periodicity measure
26. BP δP stability measure
27. BP correlation measure
28. Absence of BP peaks
29. PPG periodicity measure
30. PPG δP stability measure
31. PPG correlation Measure
32. Absence of PPG peaks

Supplementary Figure 9: VF – Importance of Features. Importance of features for classification of VF alarms calculated with the random forest algorithms.

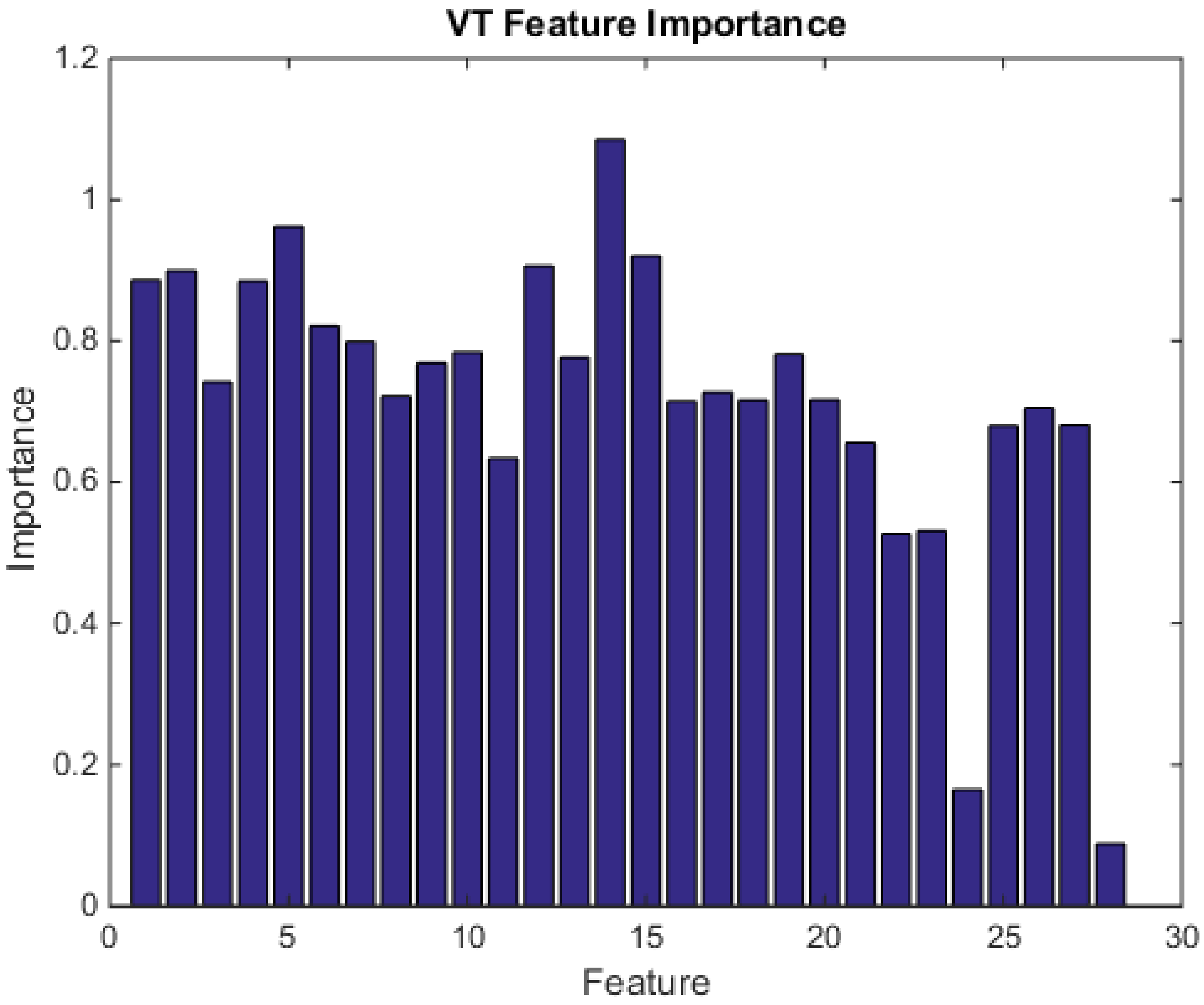

1. ECG 1 meet VT criteria
2. ECG 1 periodicity measure
3. ECG 1 sharpness measure
4. ECG 1 correlation measure
5. ECG 1 peak height stability measure
6. ECG 1 complexity measure
7. ECG 1 minimum bandwidth
8. ECG 1 max power to total power ratio
9. ECG 1 number of peaks with normalized power of at least 0.2
10. ECG 1 heart rate for segment
11. ECG 2 meet VT criteria
12. ECG 2 periodicity measure
13. ECG 2 sharpness measure
14. ECG 2 correlation measure
15. ECG 2 peak height stability measure
16. ECG 2 complexity measure
17. ECG 2 minimum bandwidth
18. ECG 2 max power to total power ratio
19. ECG 2 number of peaks with normalized power of at least 0.2
20. ECG 2 heart rate for segment
21. BP periodicity measure
22. BP δP stability measure
23. BP correlation measure
24. BP decreasing δP
25. PPG periodicity measure
26. PPG δP stability measure
27. PPG correlation measure
28. PPG decreasing δP

Supplementary Figure 10: VT – Importance of Features. Importance of features for classification of VT alarms calculated with the random forest algorithms.

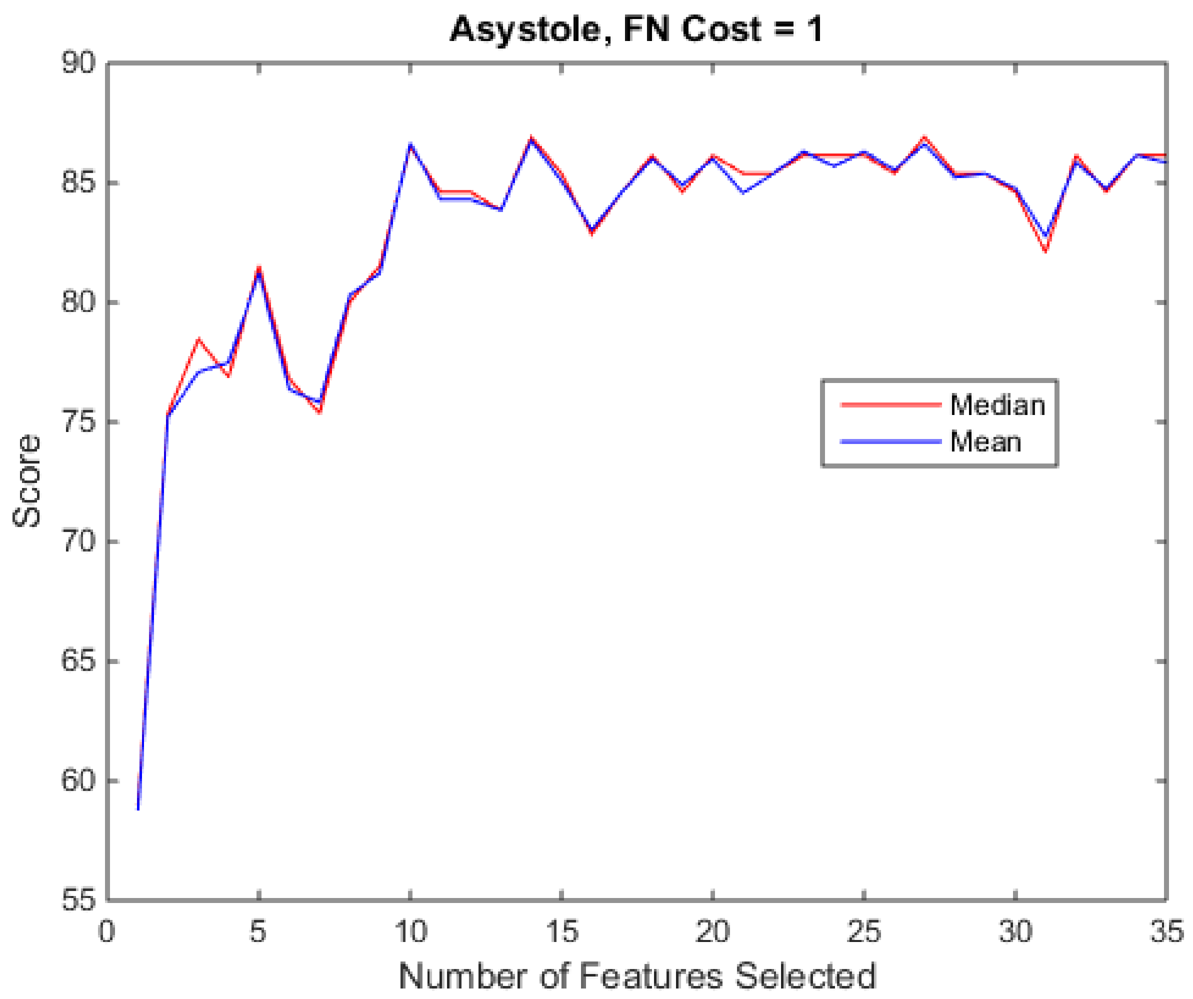

Supplementary Figure 11: Asystole – Score vs Number of Features Selected. Median and mean score curves vs number of features selected for asystole over 5 runs.

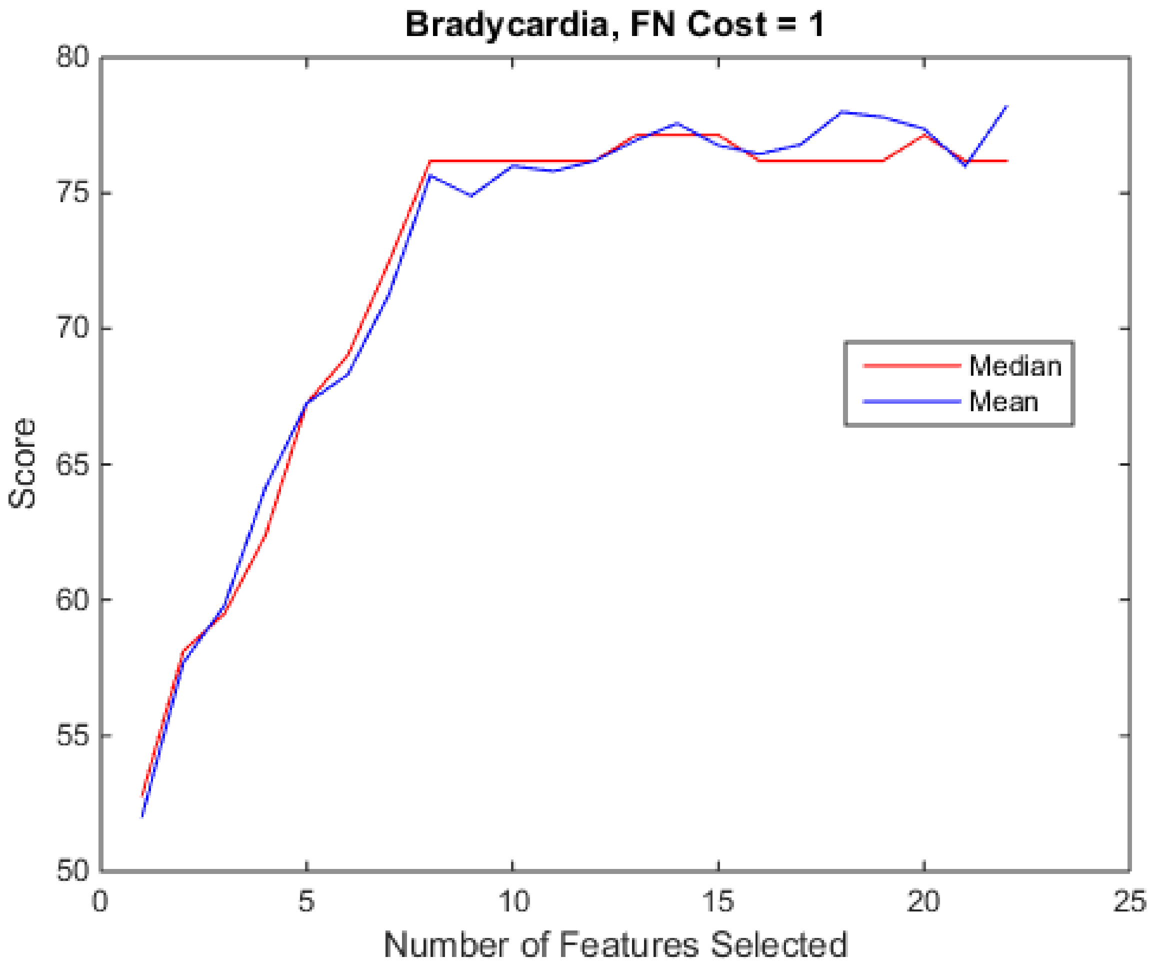

Supplementary Figure 12: Bradycardia – Score vs Number of Features Selected. Median and mean score curves vs number of features selected for bradycardia over 5 runs.

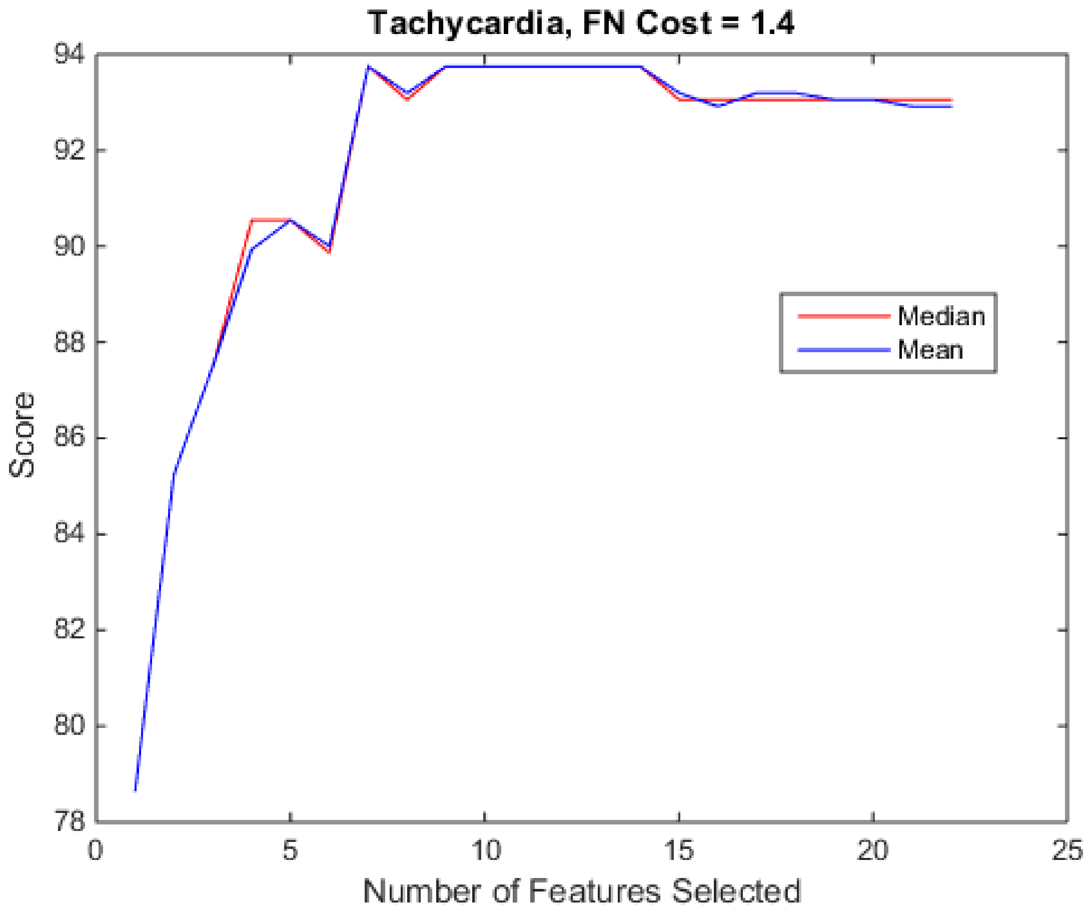

Supplementary Figure 13: Tachycardia – Score vs Number of Features Selected. Median and mean score curves vs number of features selected for tachycardia over 5 runs.

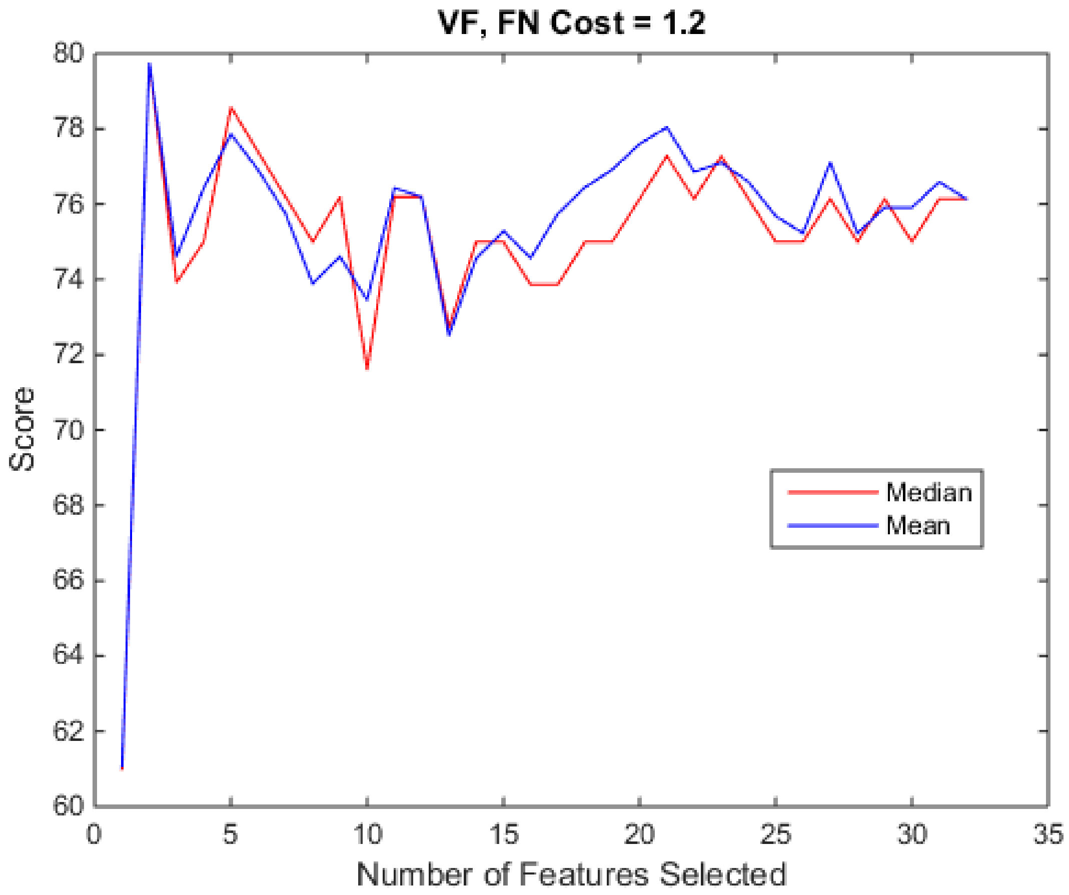

Supplementary Figure 14: VF – Score vs Number of Features Selected. Median and mean score curves vs number of features selected for VF over 5 runs.

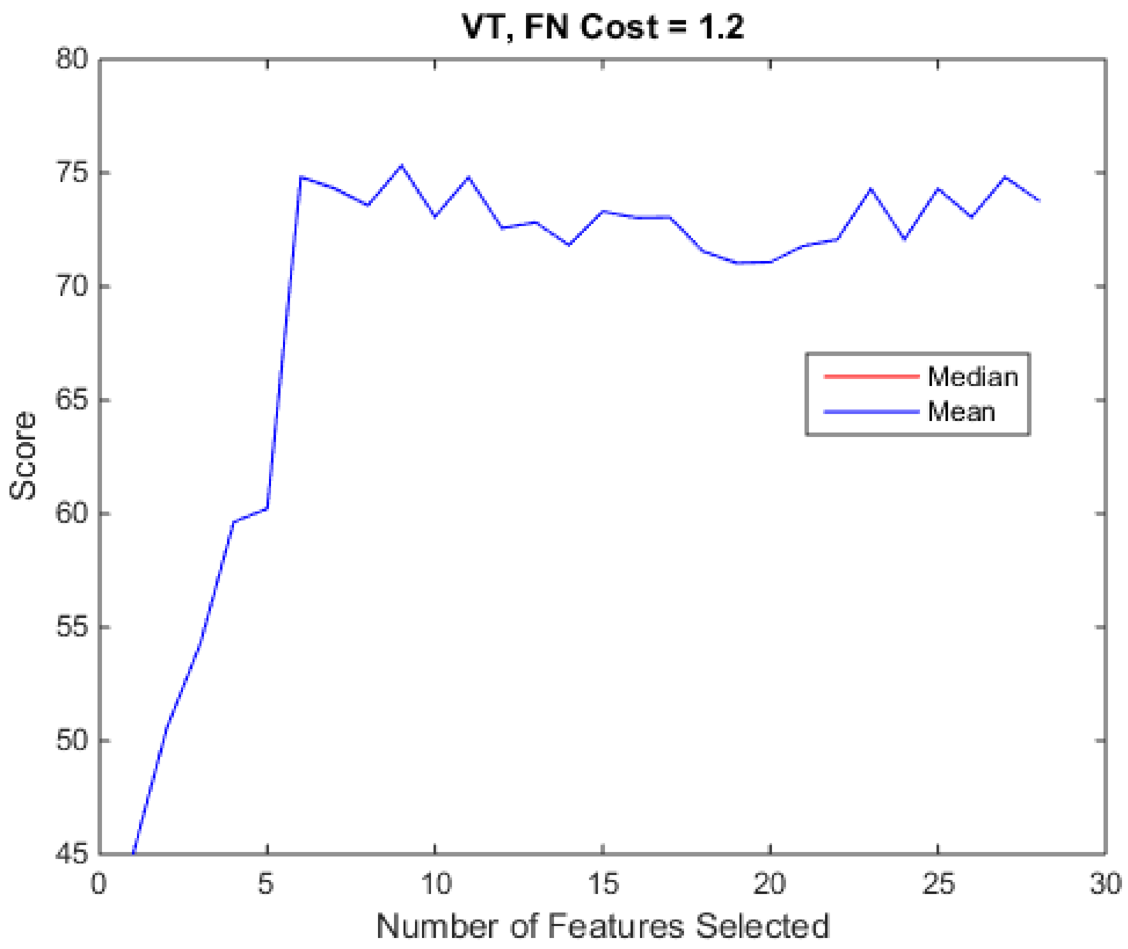

Supplementary Figure 15: VT – Score vs Number of Features Selected. Median and mean score curves vs number of features selected for VT over 5 runs.

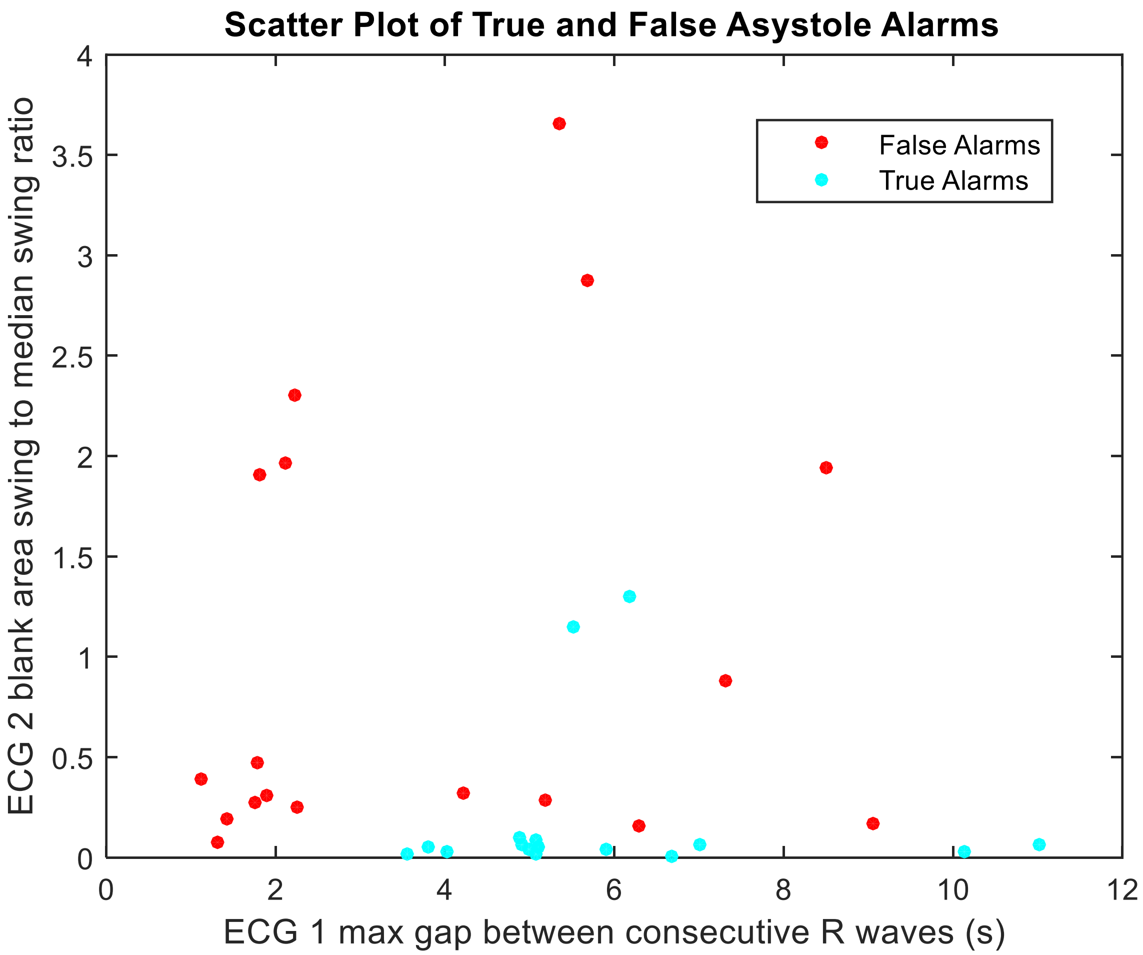

Supplementary Figure 16: Asystole – Scatter Plot. Scatter plot of true and false asystole alarms with the two most important features, ECG 1 maximum gap between consecutive R peaks and ECG 2 blank area swing to median swing ratio.

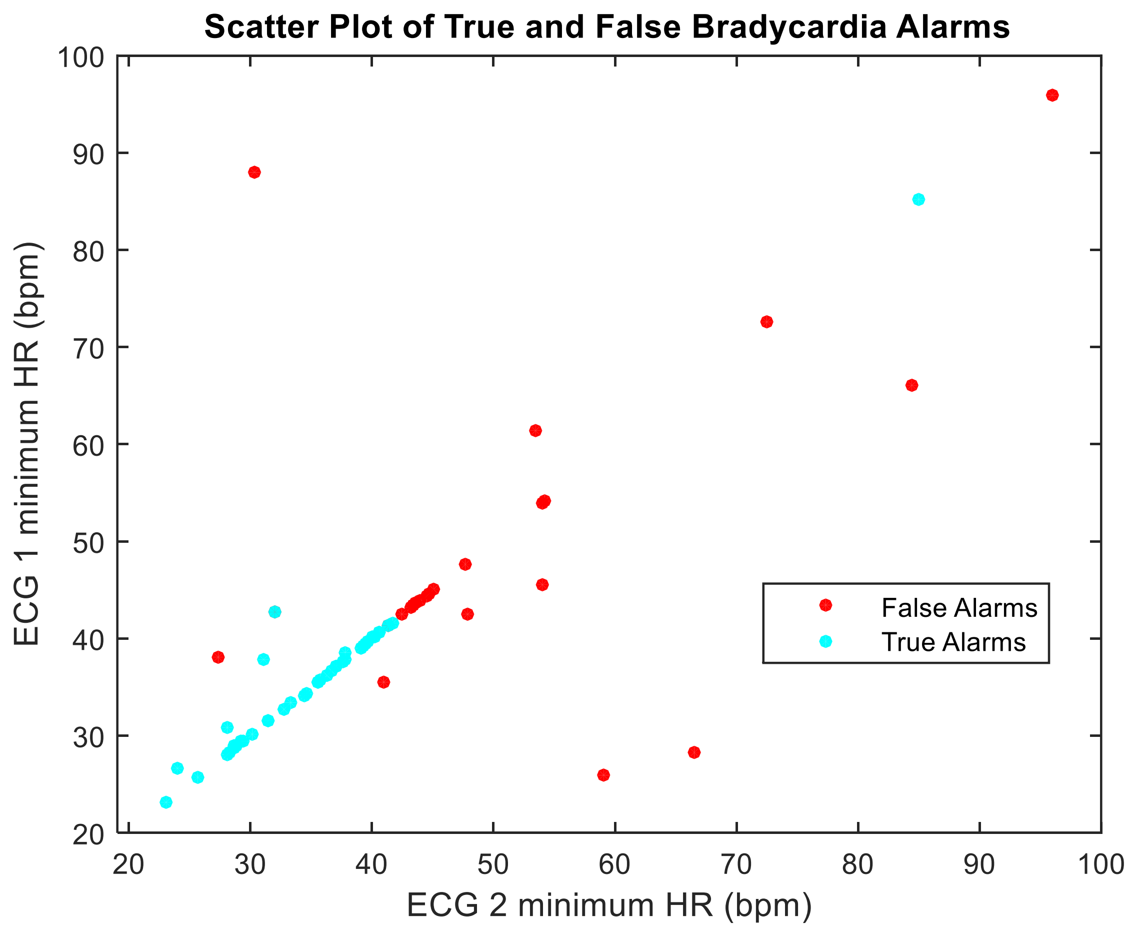

Supplementary Figure 17: Bradycardia – Scatter Plot. Scatter plot of true and false bradycardia alarms with the two most important features, ECG 2 minimum heart rate (HR) and ECG 1 minimum heart rate (HR).

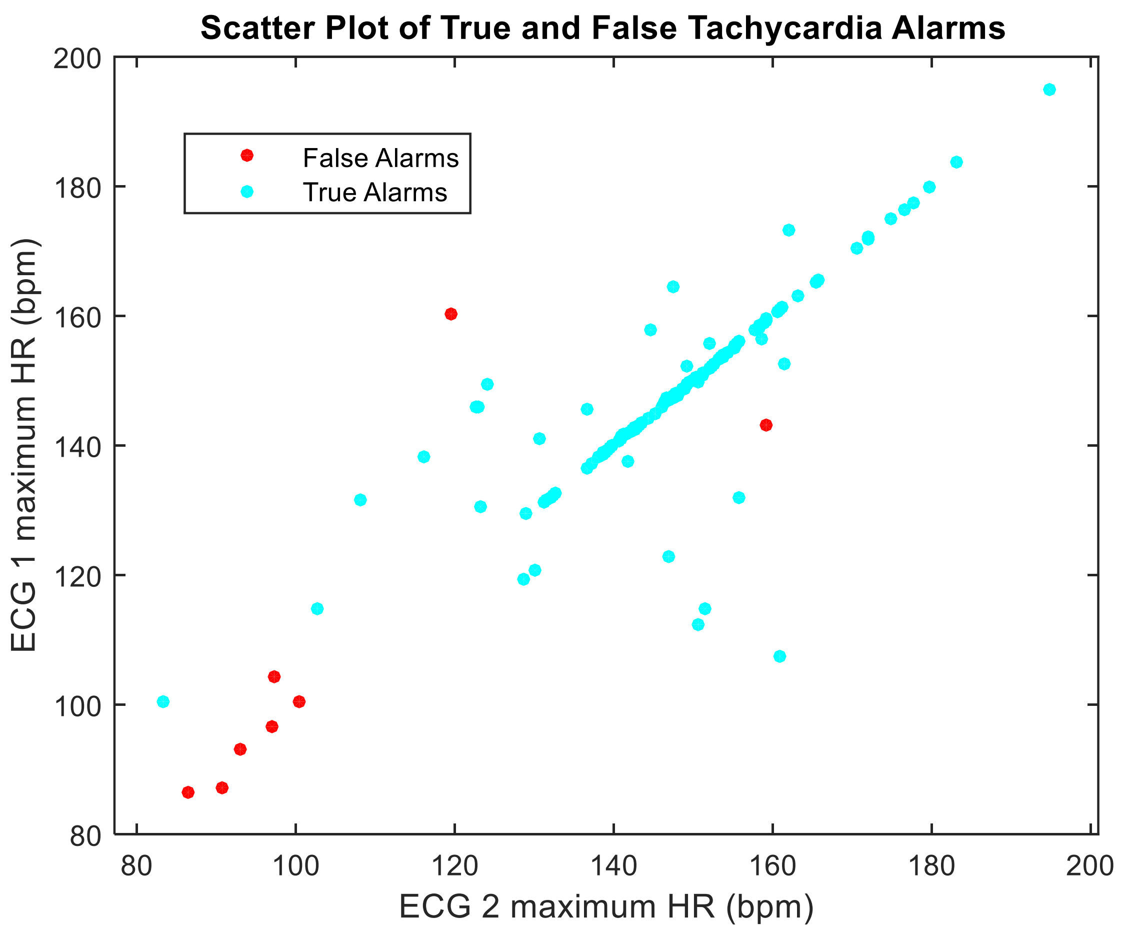

Supplementary Figure 18: Tachycardia – Scatter Plot. Scatter plot of true and false tachycardia alarms with the two most important features, ECG 2 maximum heart rate (HR) and ECG 1 maximum heart rate (HR).

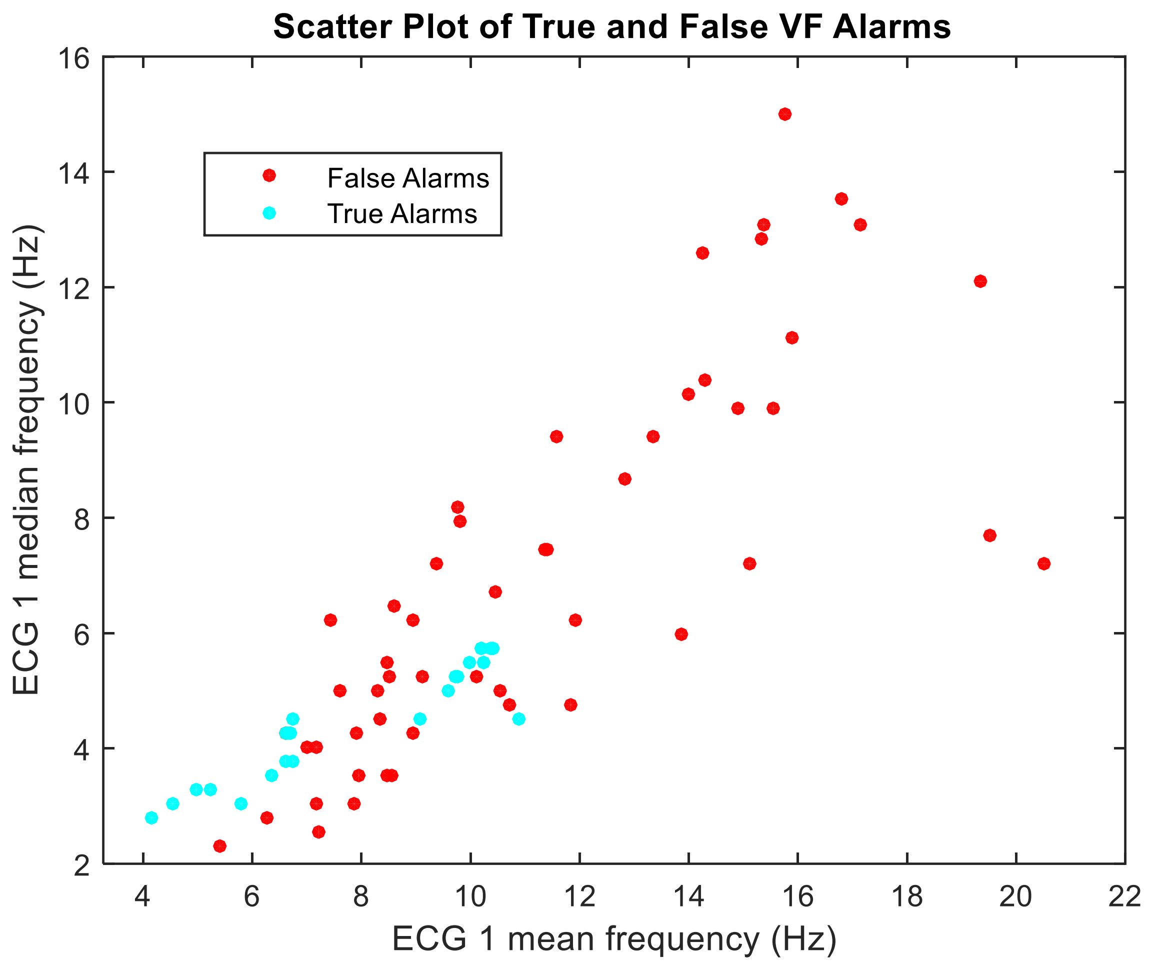

Supplementary Figure 19: VF – Scatter Plot. Scatter plot of true and false VF alarms with the two most important features, ECG 1 mean frequency and ECG 1 median frequency.

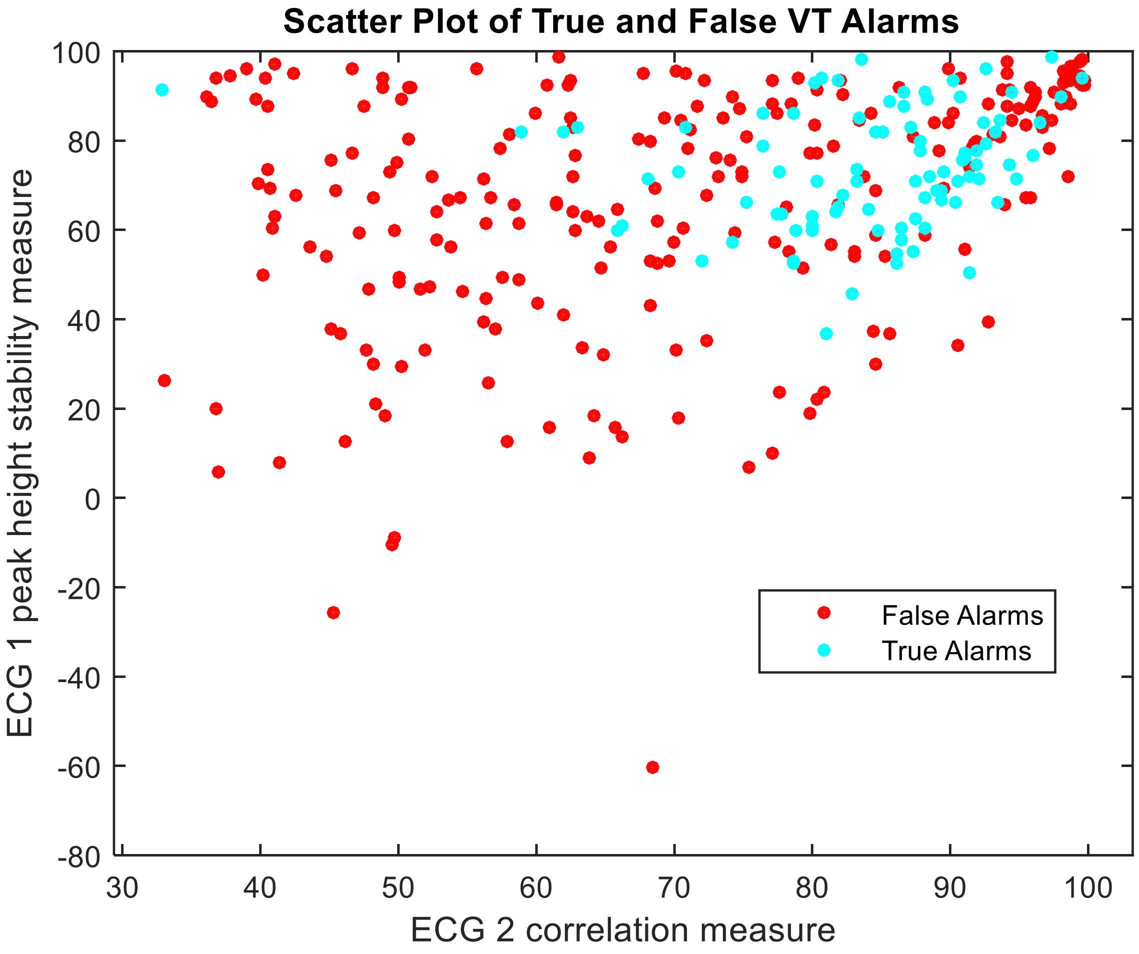

Supplementary Figure 20: VT – Scatter Plot. Scatter plot of true and false VT alarms with the two most important features, ECG 2 correlation measure and ECG 1 peak height stability measure.

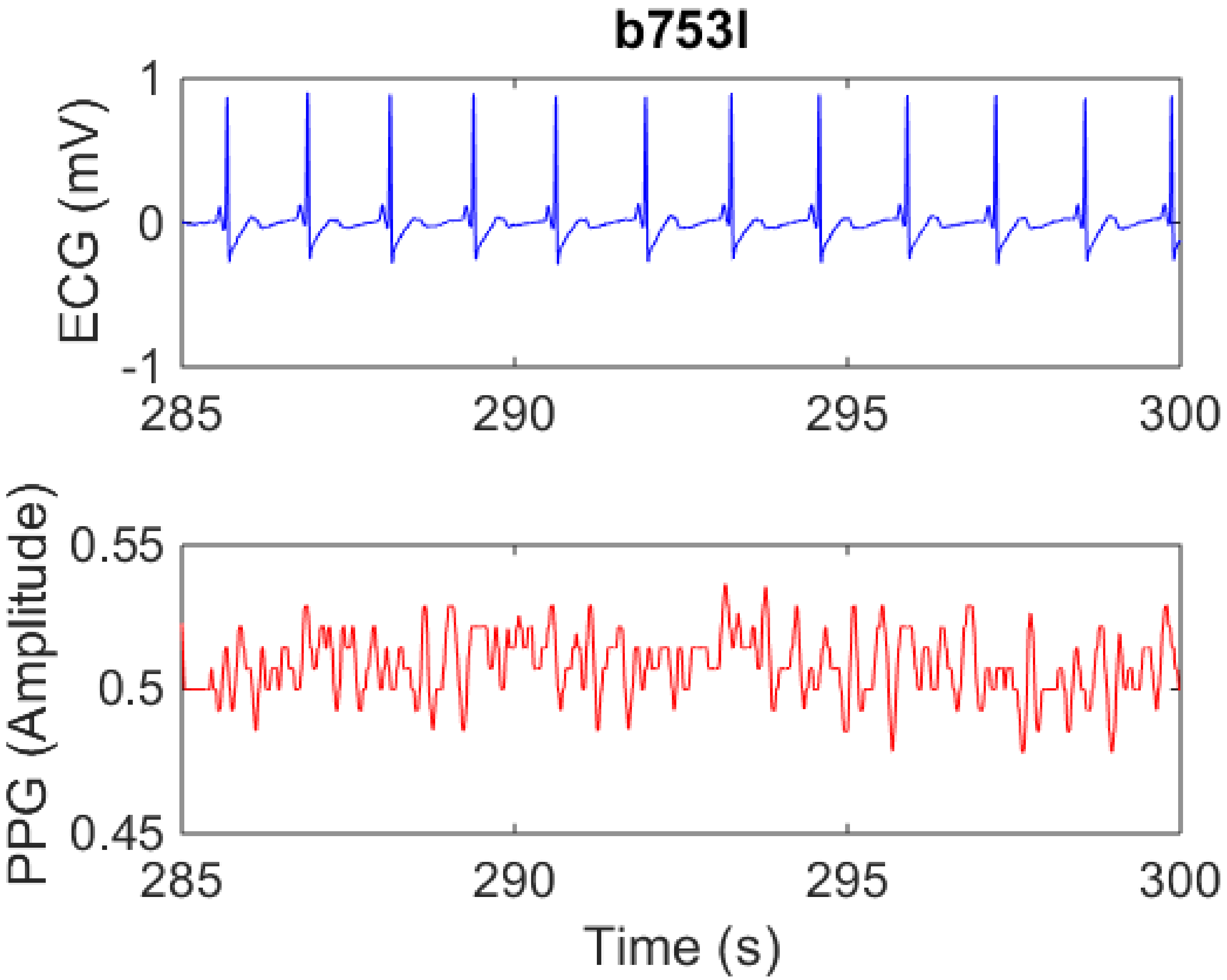

Supplementary Figure 21: Illustration of Picking the Cleanest Signal in Bradycardia. For bradycardia alarms, the signal with the highest correlation measure is selected for the use of classification of the alarm. For this particular record b753l, the ECG signal is selected because the ECG signal has a correlation measure of 99.5 while the PPG has a correlation measure of 54.7.

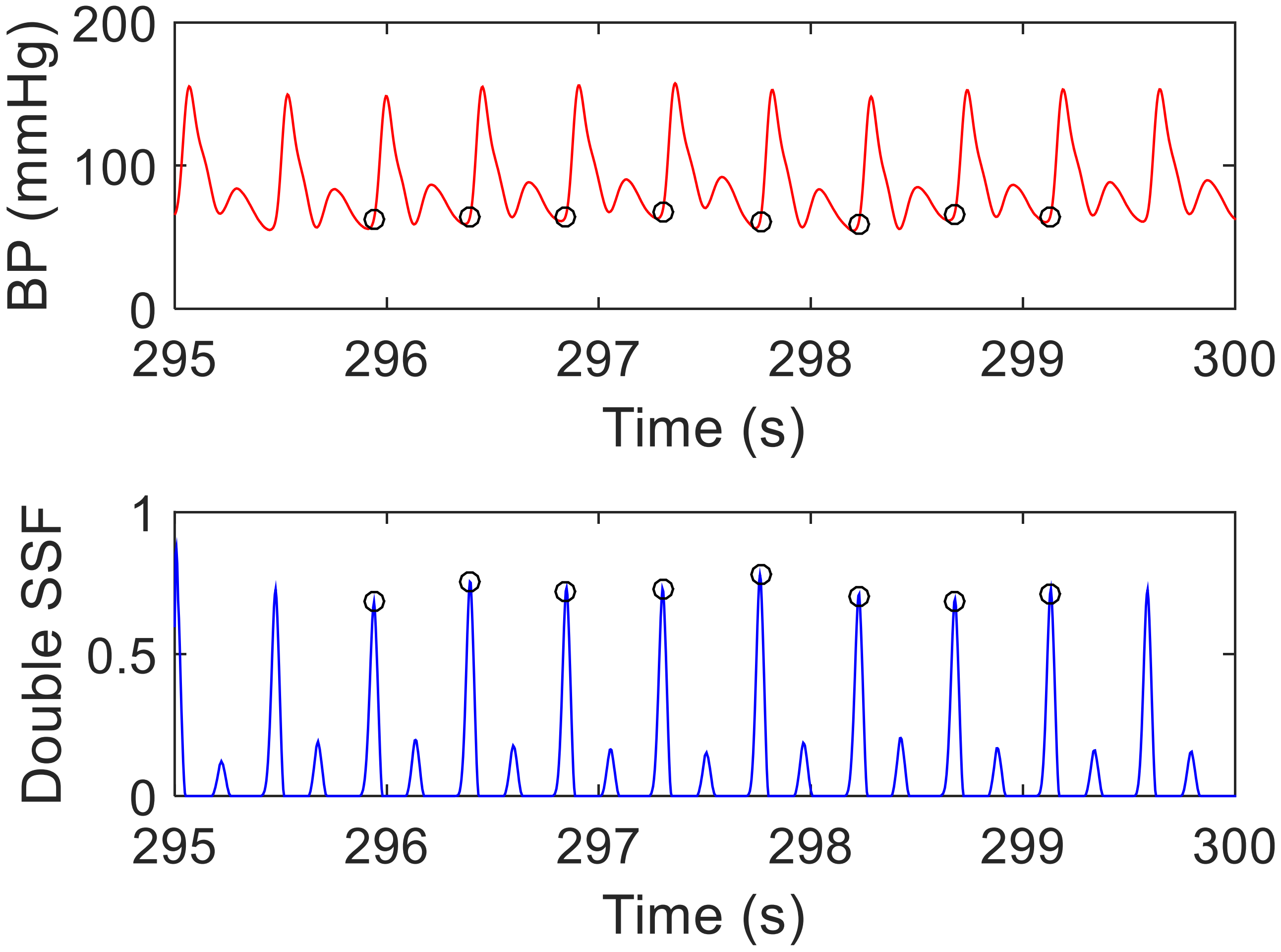

Supplementary Figure 22: Detection of Onset of Waveforms in ABP/PPG. A modified Zong's method for identification of valleys in BP and PPG was used. The slope sum function was calculated twice on the BP or PPG signal. The double SSF would have sharp peaks at where the valleys are at the original signals. Then these sharp peaks in the double slope sum function are identified using the Martinez's method for QRS detection.

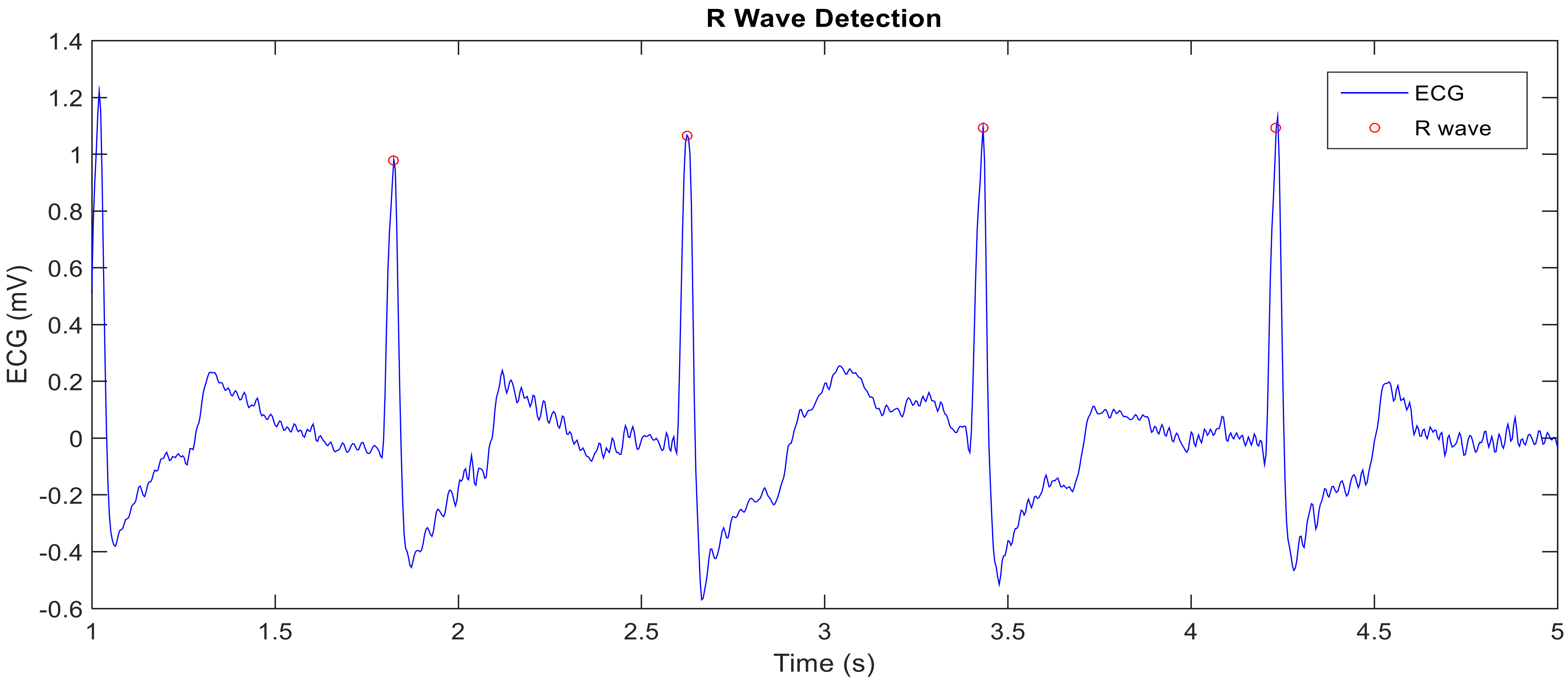

Supplementary Figure 23: R-wave Detection. The ECG signal and the R peaks detected with the ECG delineation algorithm designed by Martinez et al.

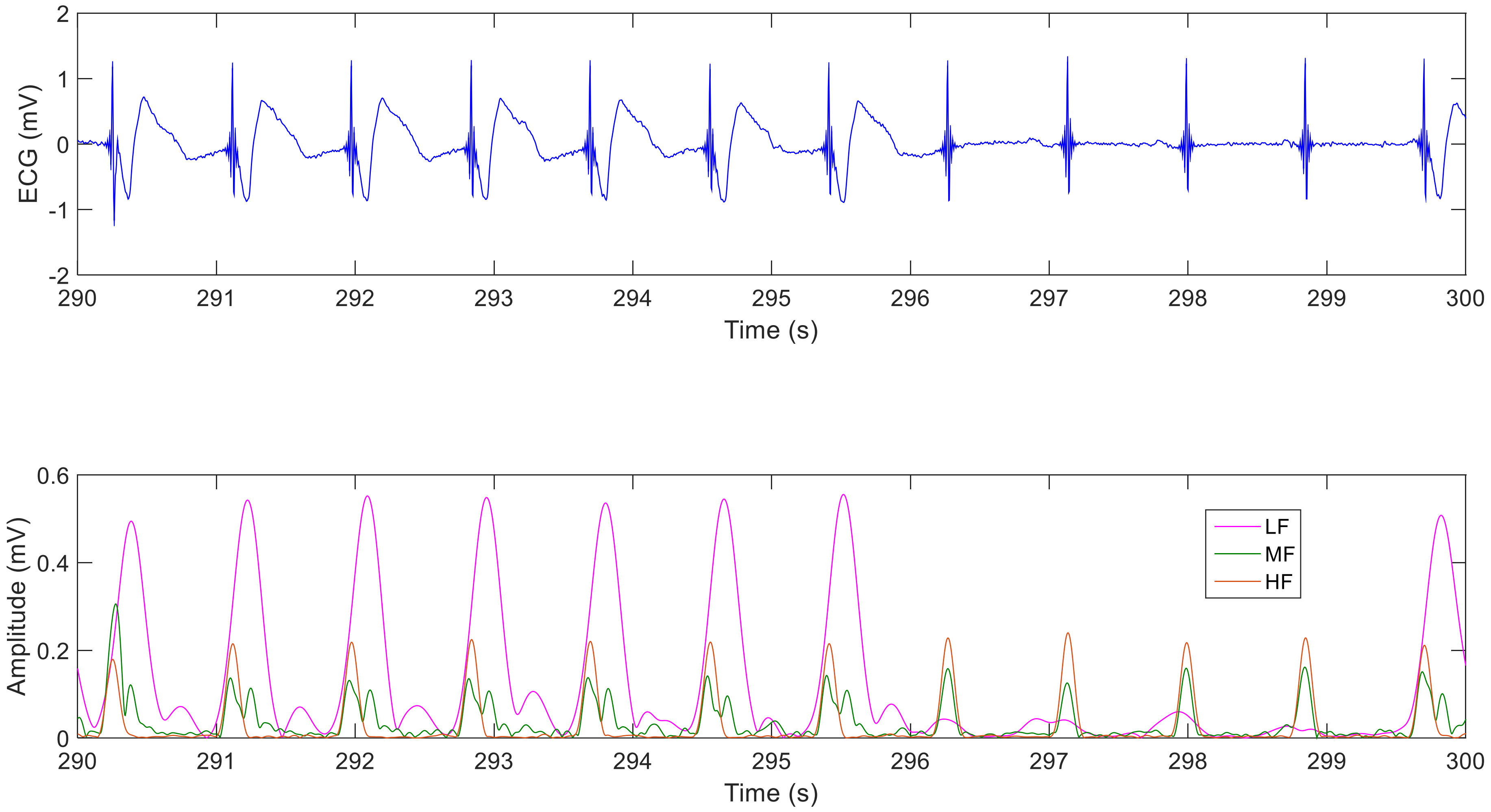

Supplementary Figure 24: Removal of Pacing Spikes. The ECG delineation algorithm could misclassify pacing spikes as R peaks. The method of amplitude envelope was used to remove false R peak detection due to pacing spikes. R peaks detected at the locations where the amplitude envelope of HF is higher than 0.1mV are removed.

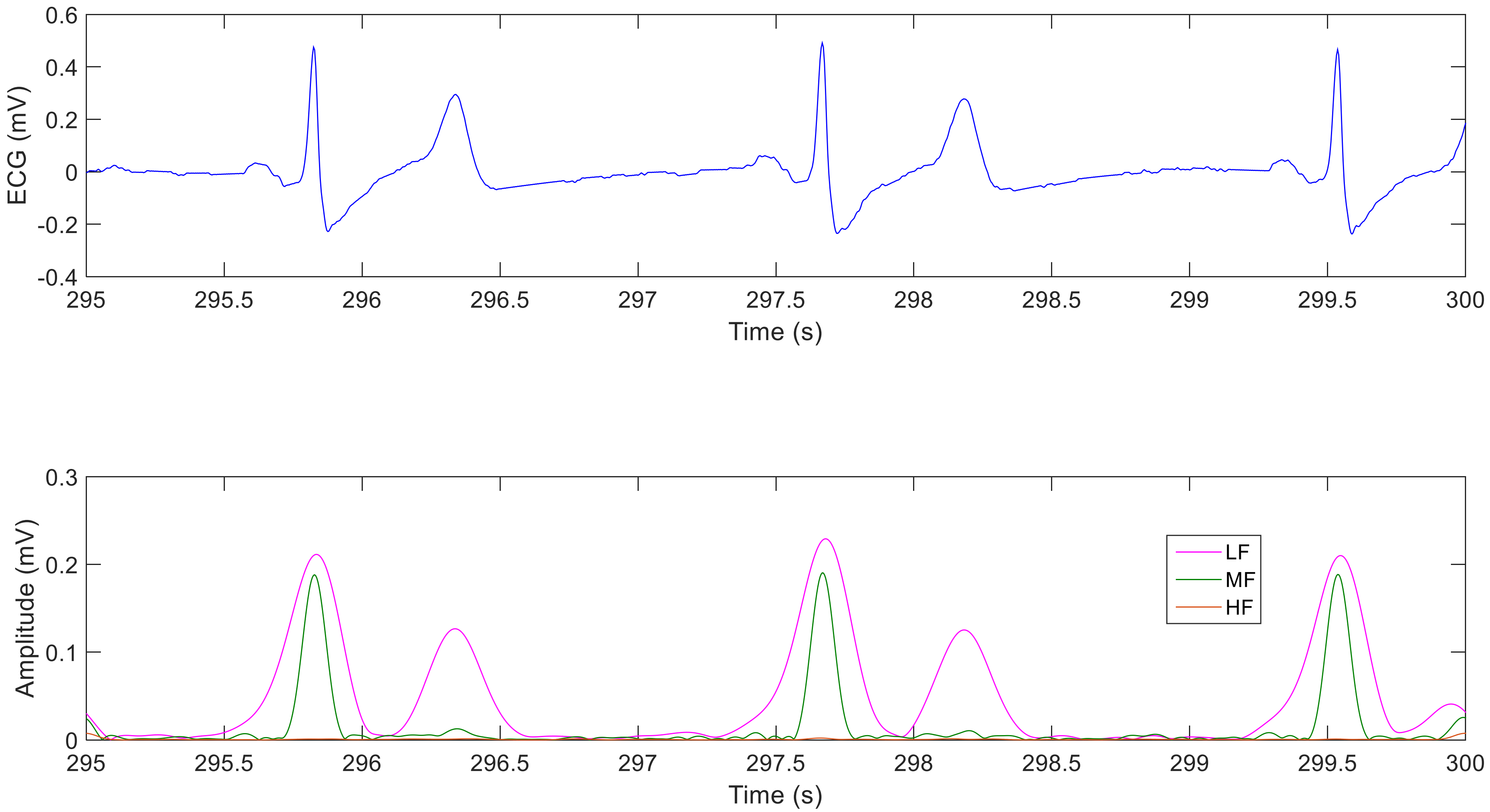

Supplementary Figure 25: Removal of T-wave Oversensing. For bradycardia alarms, the ECG delineation algorithm may misclassify T waves as R peaks. Therefore, false R peak detection due to T-wave over-sensing is removed by using the method of amplitude envelope. The amplitude envelope of MF is much higher at the R peaks than at the T waves. Therefore, the beat annotations at the locations where the amplitude envelope of MF is below 0.05 would be removed.

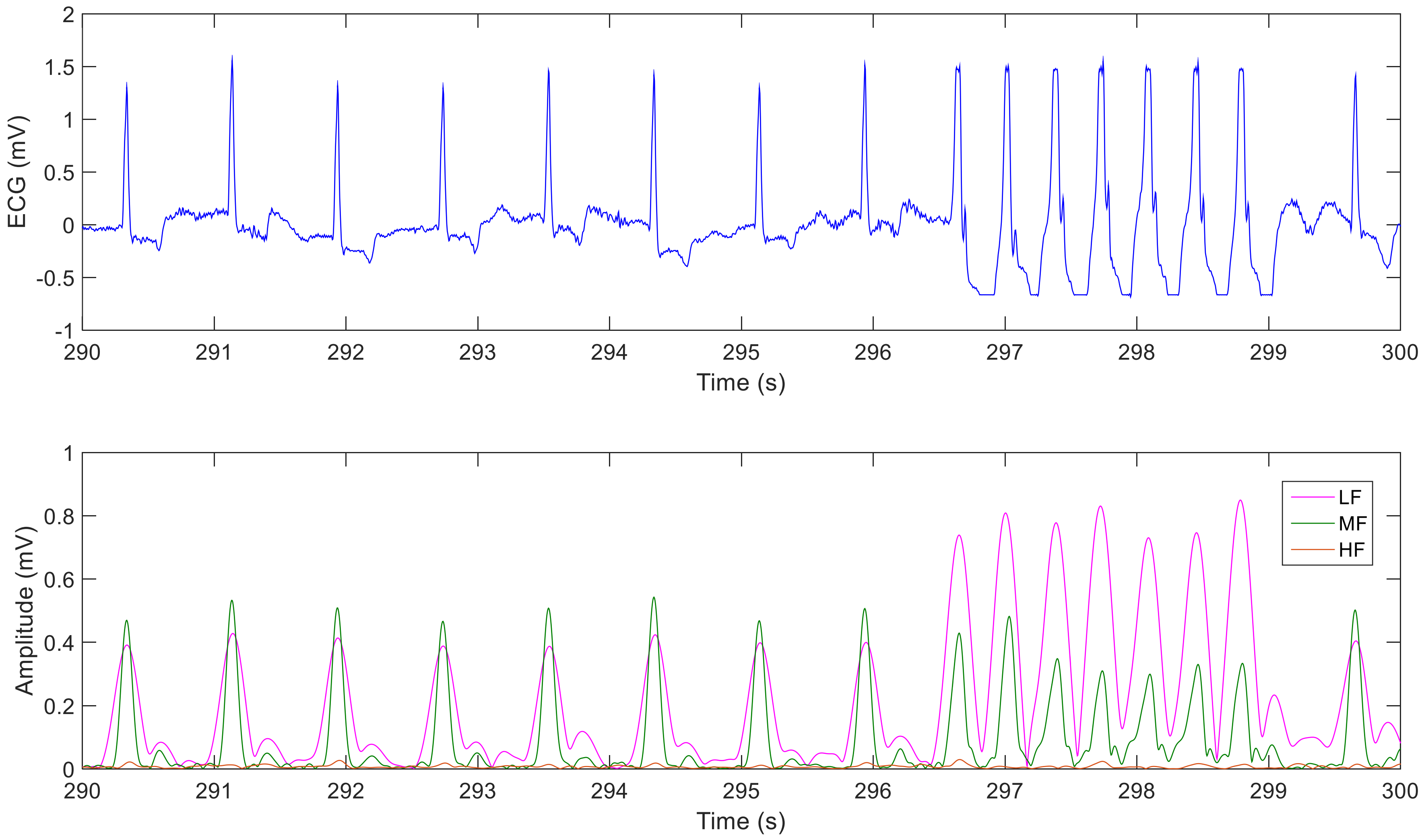

Supplementary Figure 26: Identification of Ventricular Beats. For VT alarms, beats are labelled as ventricular tachycardia beats if the amplitude envelope of LF is greater than the difference between the amplitude envelope of MF and the amplitude envelope of HF. There are ventricular beats within 296.5s and 299s as shown.

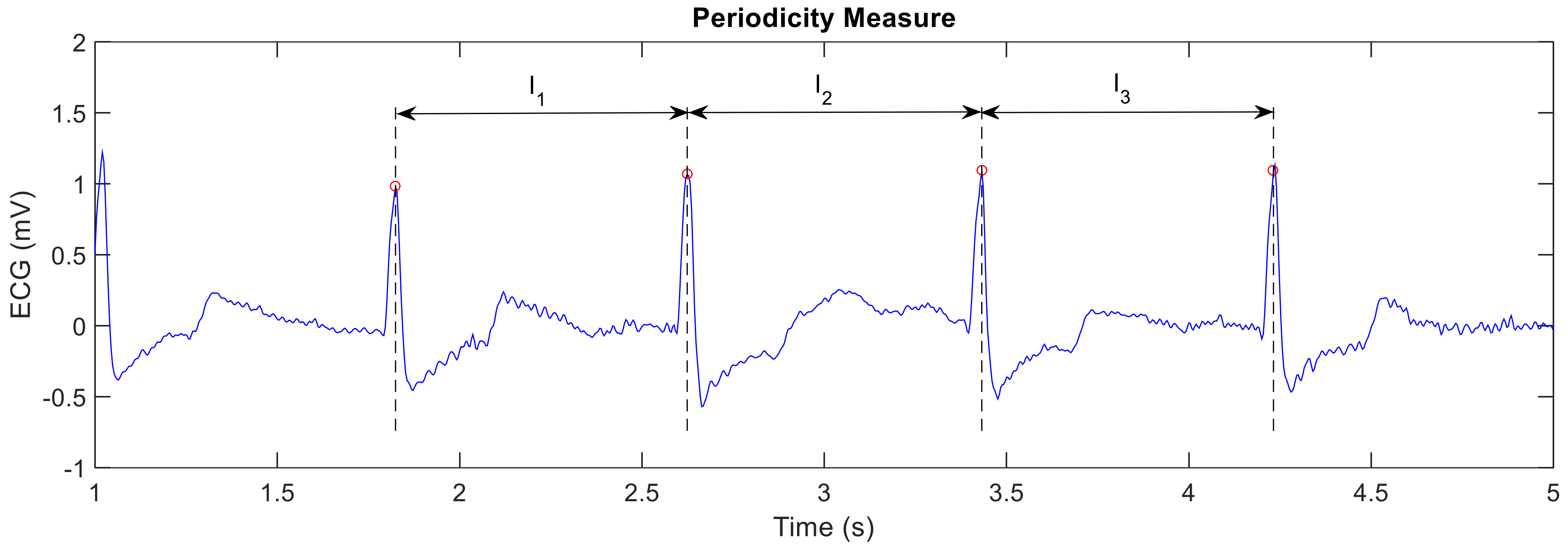

Supplementary Figure 27: ECG – Periodicity Measure. Each interval, I_i, is calculated as the time difference between consecutive R peaks.

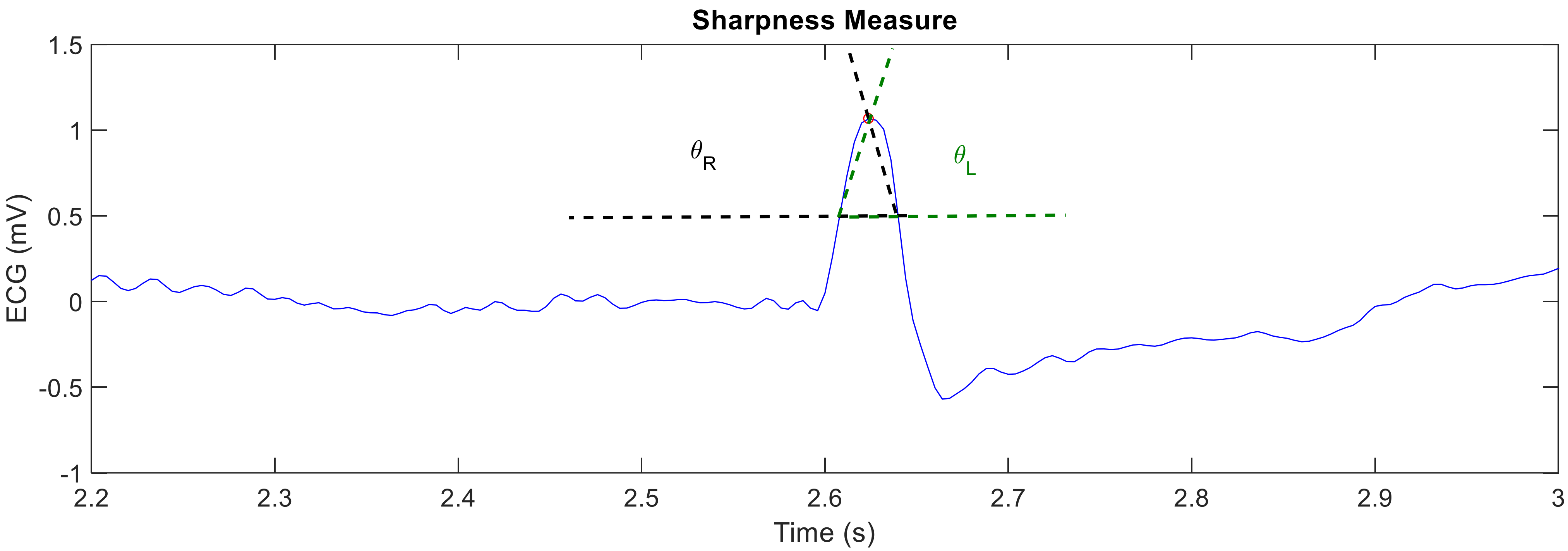

Supplementary Figure 28: ECG – Sharpness Measure. The less of θ_L and θ_R is defined as the sharpness, S, of the QRS complex.

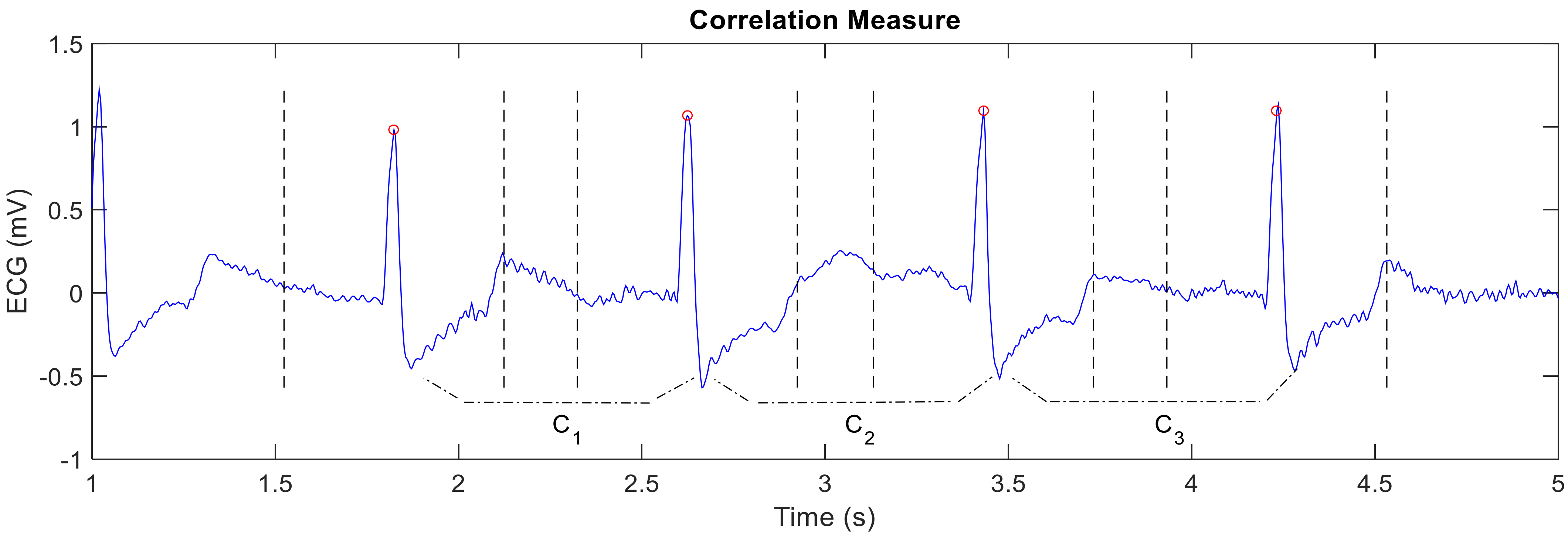

Supplementary Figure 29: ECG – Correlation Measure. Correlations between successive beats, C_i's, are calculated. Each window is centered at the R peak. The window begins at 300ms before the R peak and ends at 300ms after the R peak.

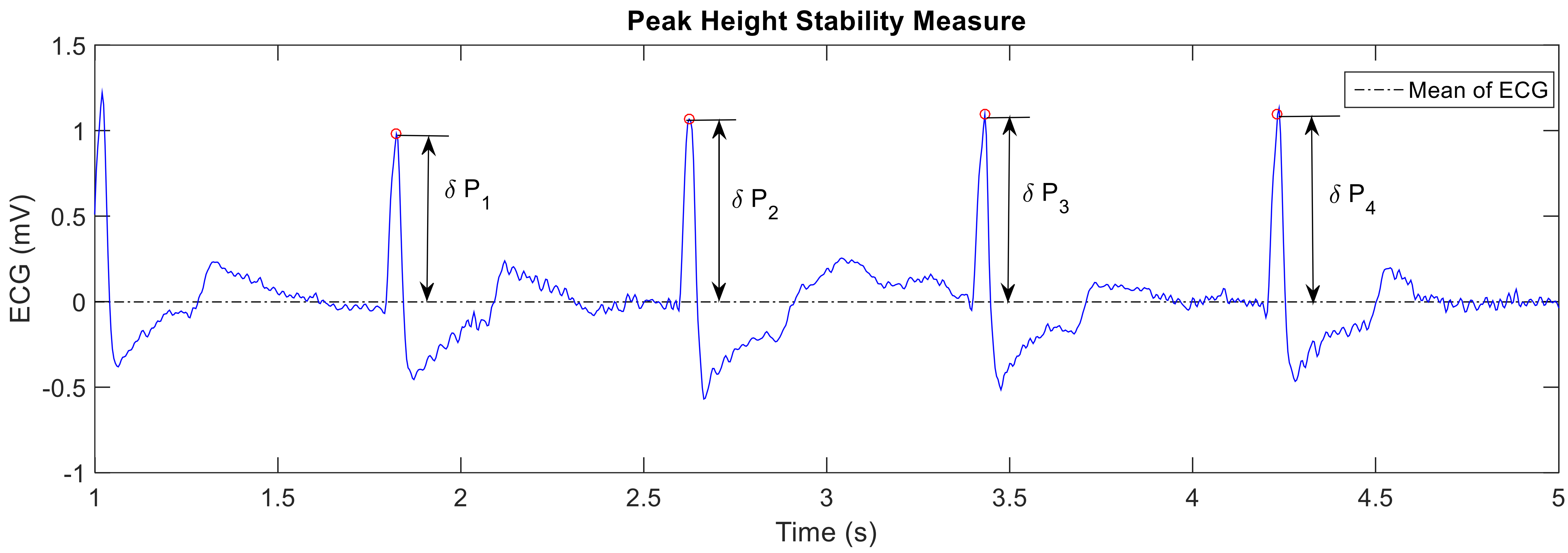

Supplementary Figure 30: ECG – Peak Height Stability Measure. Each peak height, δP_i, is calculated as the difference between the amplitude of the signal at the peak and the mean amplitude of the ECG signal.

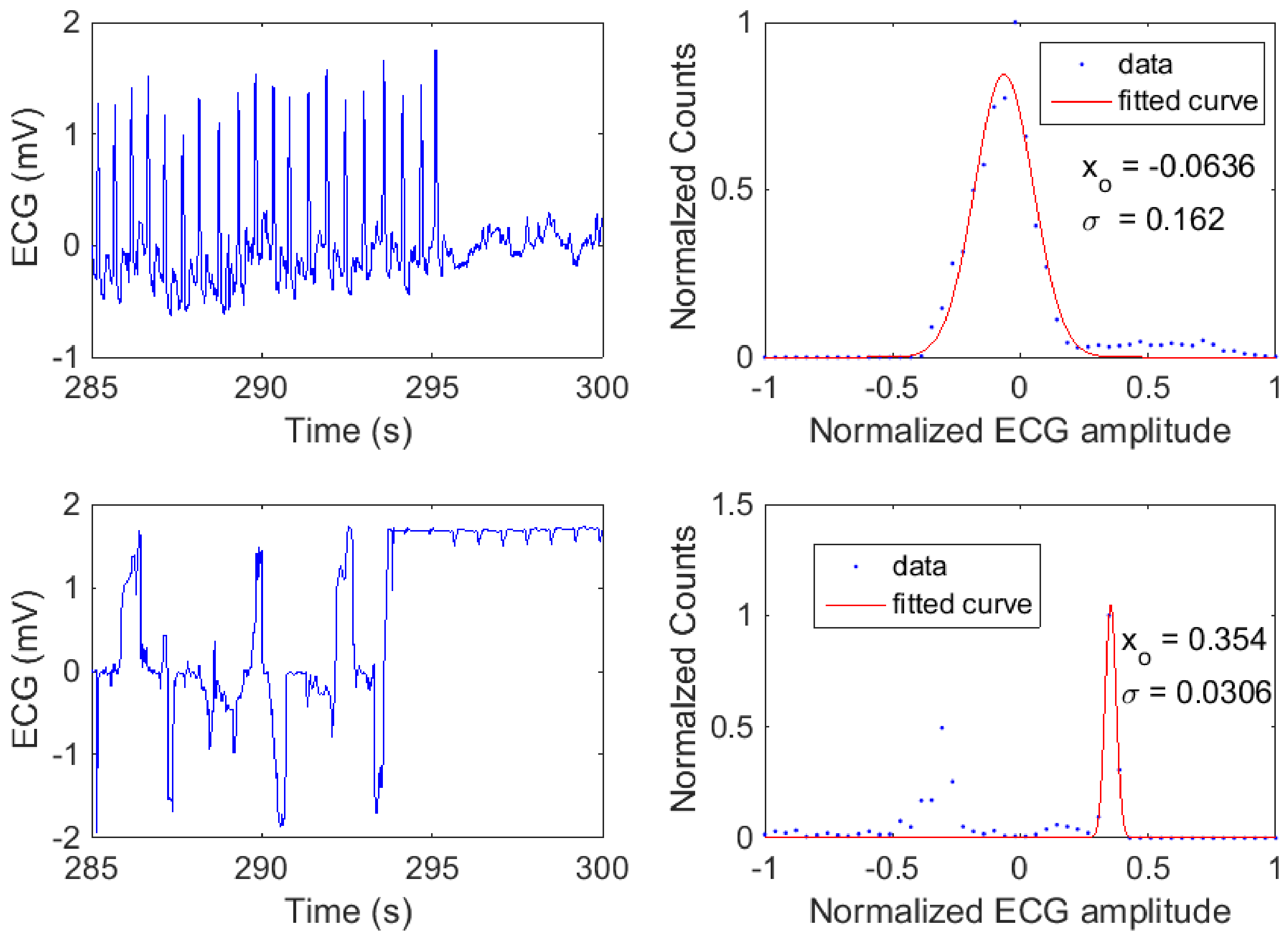

Supplementary Figure 31: ECG – Histogram. The top-left panel shows an ECG signal with good quality while the bottom-left panel shows an ECG signal with bad quality. On their right is their respective plot of histogram of normalized ECG amplitude with normalized count. A Gaussian distribution is fitted to each of the histograms. Good ECG signals should have a Gaussian distribution with its mean close to 0 and a fairly small standard deviation.

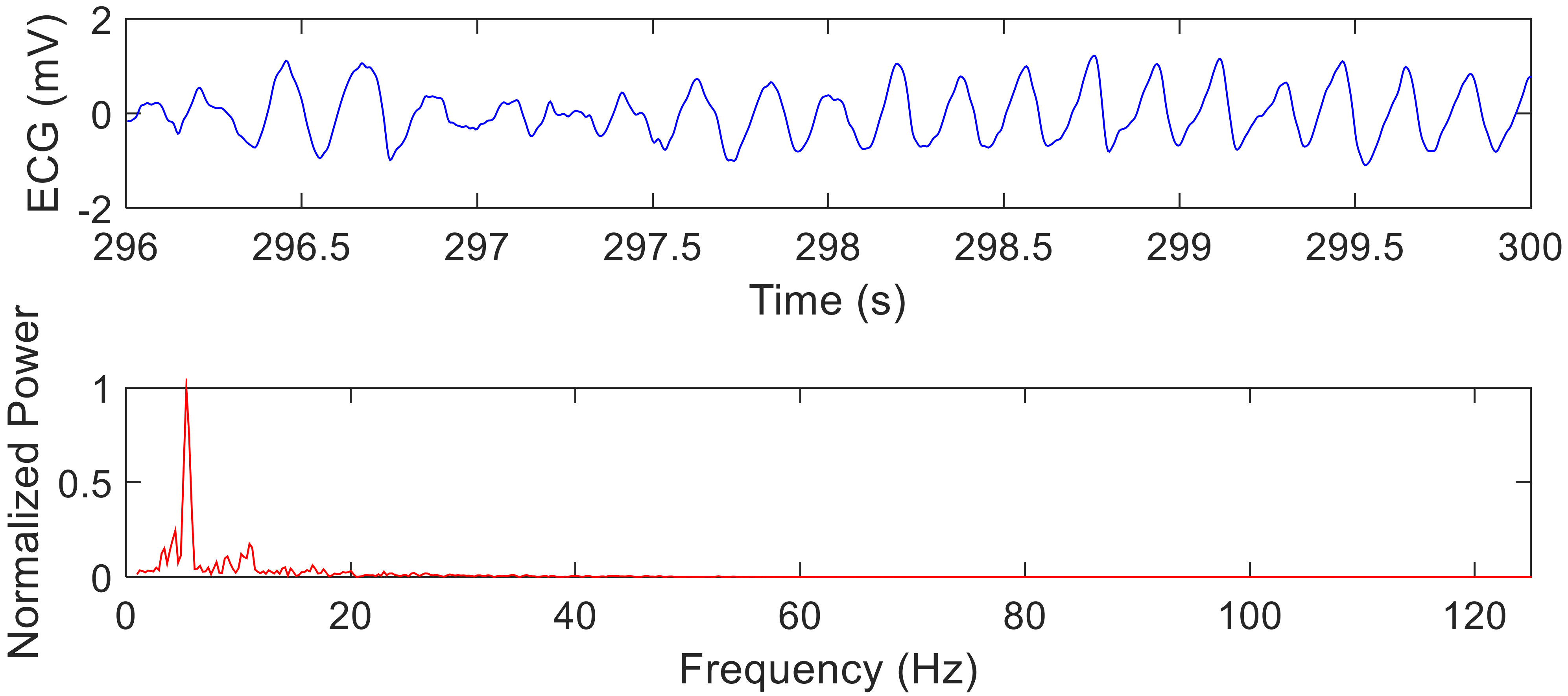

Supplementary Figure 32: Frequency Domain Analysis. Frequency domain analysis was done on VF and VT signals. Features extracted from the power spectrum include mean frequency, median frequency, maximum power to total power ratio, dominant frequency, bandwidth and number of peaks with normalized power of at least 0.2.

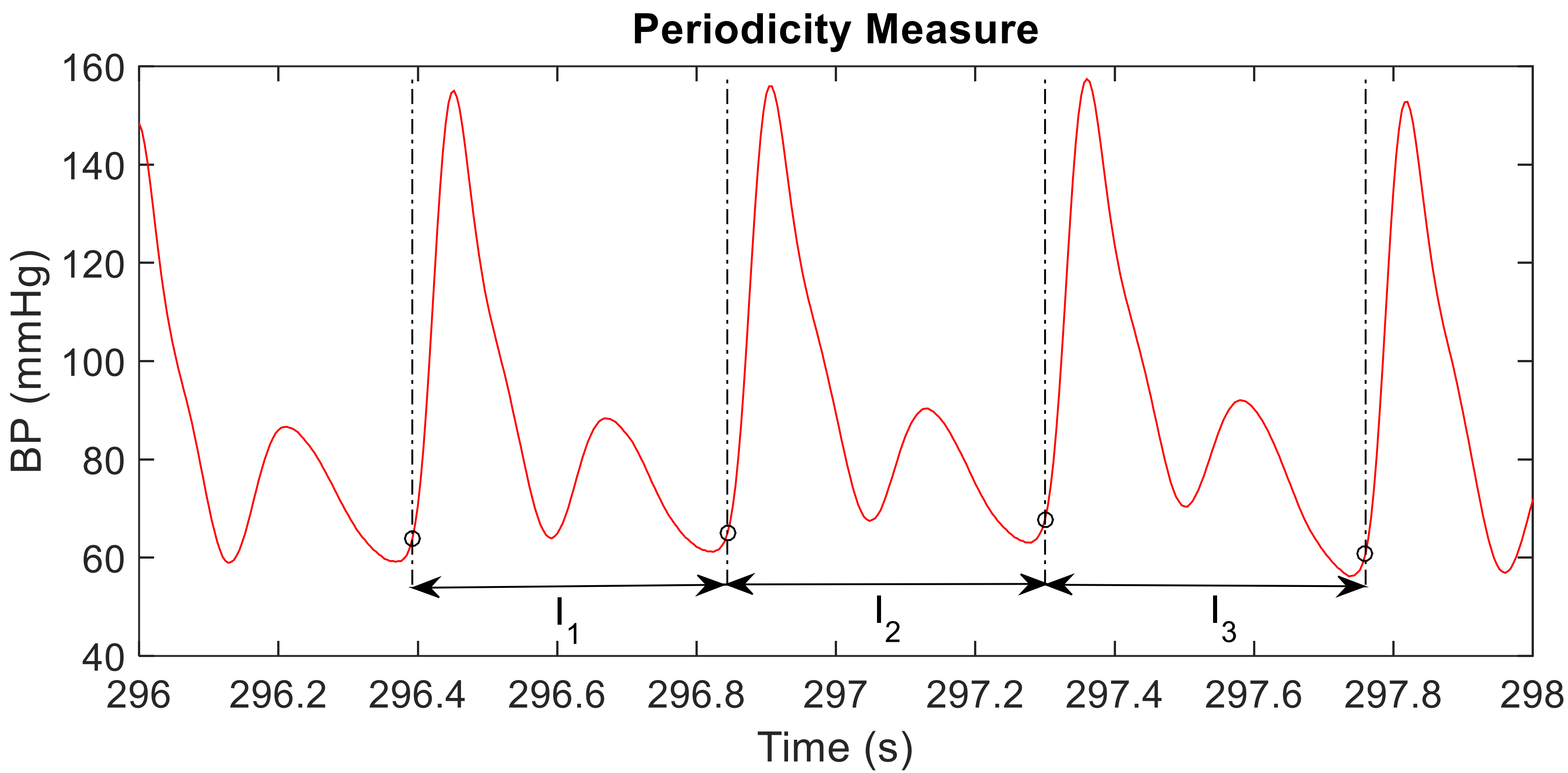

Supplementary Figure 33: ABP/PPG – Periodicity Measure. Time intervals, I = [I_1, I_2, …, I_{n-1}], between n consecutive valleys in ABP/PPG are calculated.

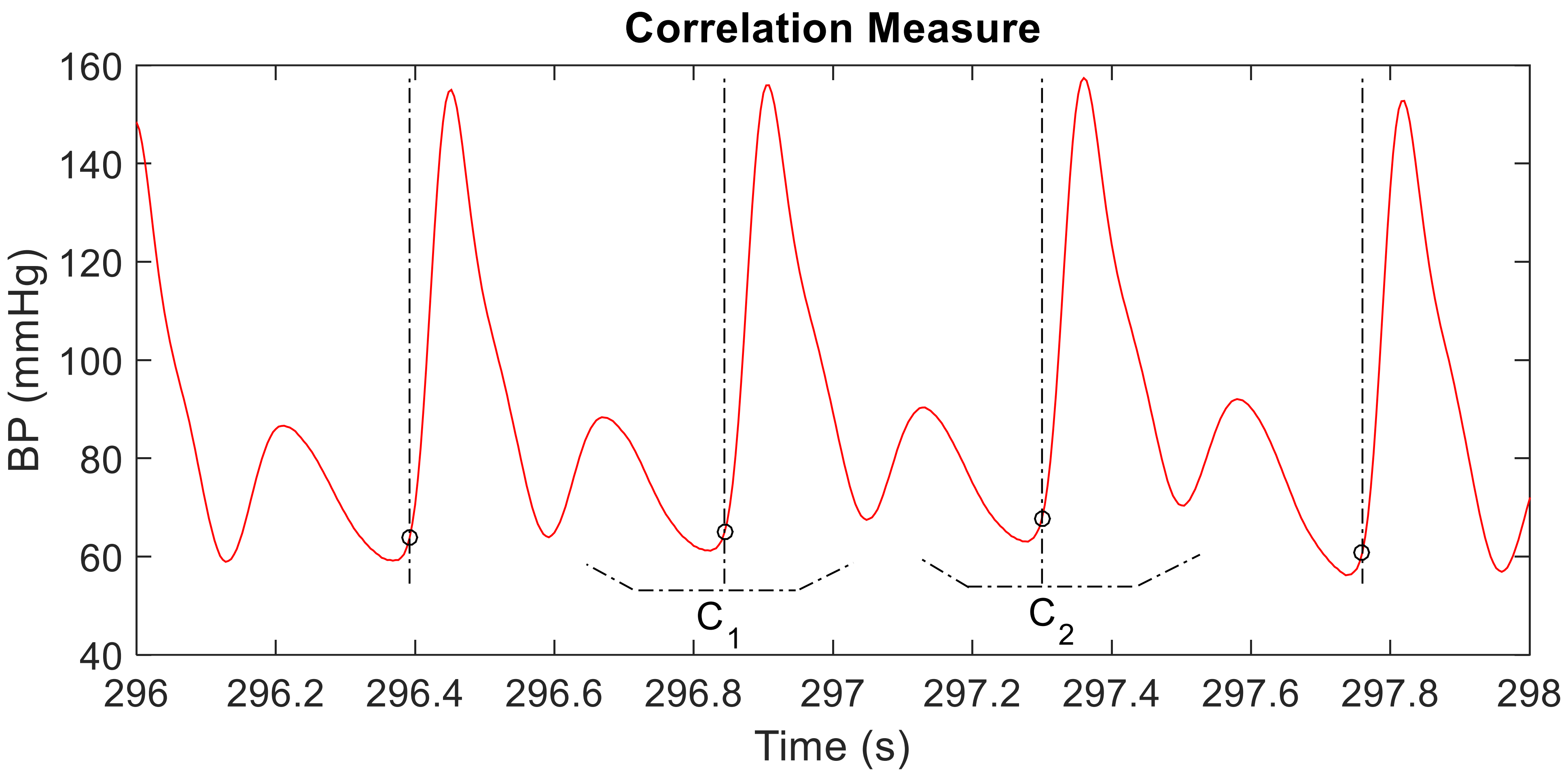

Supplementary Figure 34: ABP/PPG – Correlation Measure. Correlation between consecutive waveforms, C_i, is found.

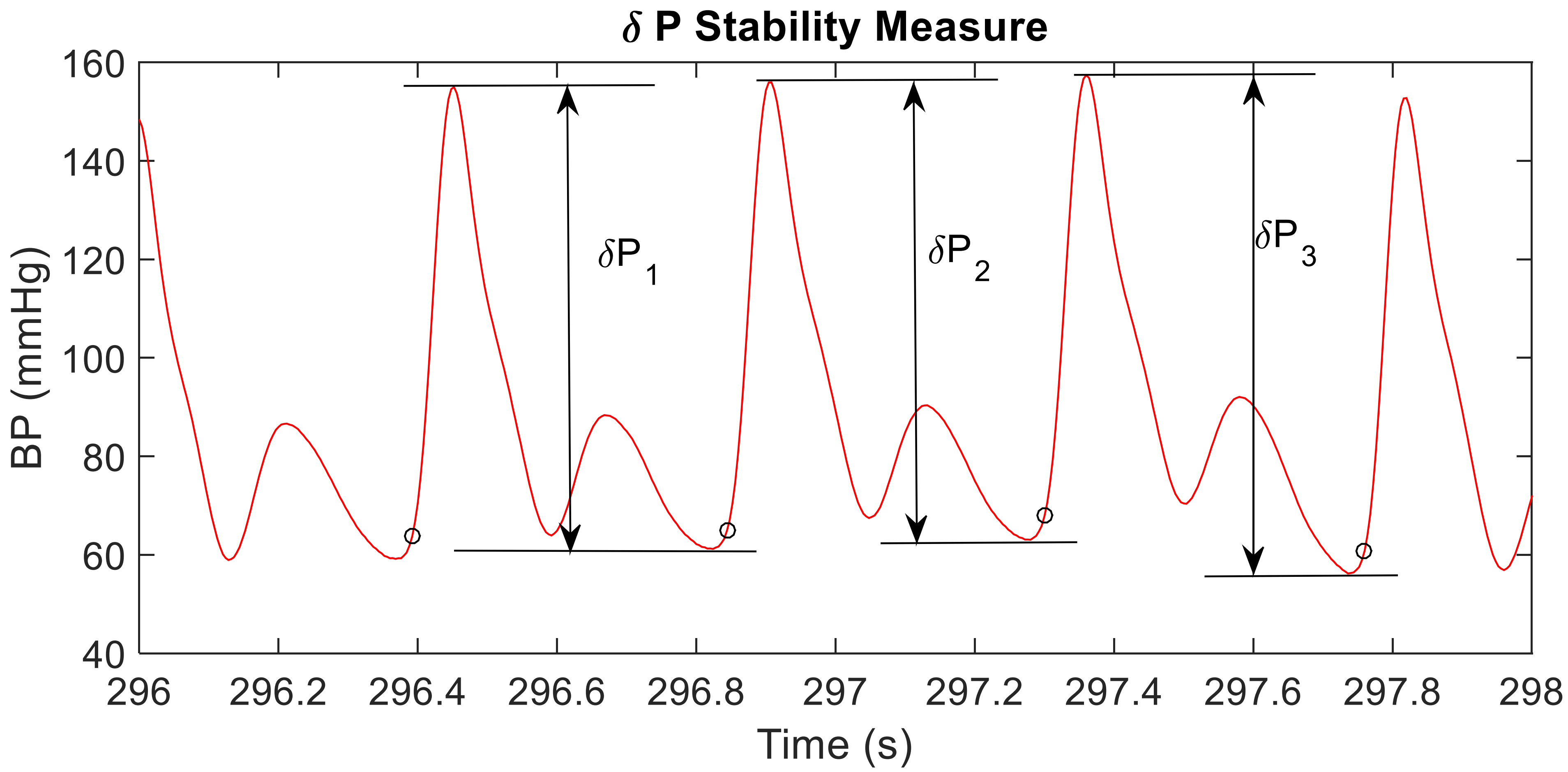

Supplementary Figure 35: ABP/PPG - δP Stability Measure. δP_i of the ith waveform is equal to the difference between the maximum value in the waveform and the minimum value in the waveform and it is stored in the vector $\delta P = [\delta P_1, \delta P_2, ..., \delta P_n]$.
